# Manometric real-time studies of the mechanochemical synthesis of zeolitic imidazolate frameworks†

**DOI:** 10.1039/c9sc05514b

**Published:** 2020-01-02

**Authors:** Ivana Brekalo, Wenbing Yuan, Cristina Mottillo, Yuneng Lu, Yuancheng Zhang, Jose Casaban, K. Travis Holman, Stuart L. James, Frédéric Duarte, P. Andrew Williams, Kenneth D. M. Harris, Tomislav Friščić

**Affiliations:** Department of Chemistry, Georgetown University 20057 Washington, D.C. USA kth7@georgetown.edu; School of Enviromental and Chemical Engineering, Foshan University Foshan 528000 China; Department of Chemistry, McGill University H3A 0B8 Montreal Quebec Canada tomislav.friscic@mcgill.ca; School of Chemistry, Queen's University Belfast BT7 1NN Belfast UK S.James@qub.ac.uk; MOF Technologies BT7 1NF Belfast UK; School of Chemistry, Cardiff University CF10 3AT Cardiff UK HarrisKDM@cardiff.ac.uk

## Abstract

We demonstrate a simple method for real-time monitoring of mechanochemical synthesis of metal–organic frameworks, by measuring changes in pressure of gas produced in the reaction. Using this manometric method to monitor the mechanosynthesis of the zeolitic imidazolate framework ZIF-8 from basic zinc carbonate reveals an intriguing feedback mechanism in which the initially formed ZIF-8 reacts with the CO_2_ byproduct to produce a complex metal carbonate phase, the structure of which is determined directly from powder X-ray diffraction data. We also show that the formation of the carbonate phase may be prevented by addition of excess ligand. The excess ligand can subsequently be removed by sublimation, and reused. This enables not only the synthesis but also the purification, as well as the activation of the MOF to be performed entirely without solvent.

## Abstract 2

We demonstrate a simple method for real-time monitoring of mechanochemical synthesis of metal–organic frameworks, by measuring changes in pressure of gas produced in the reaction.

## Graphical abstract

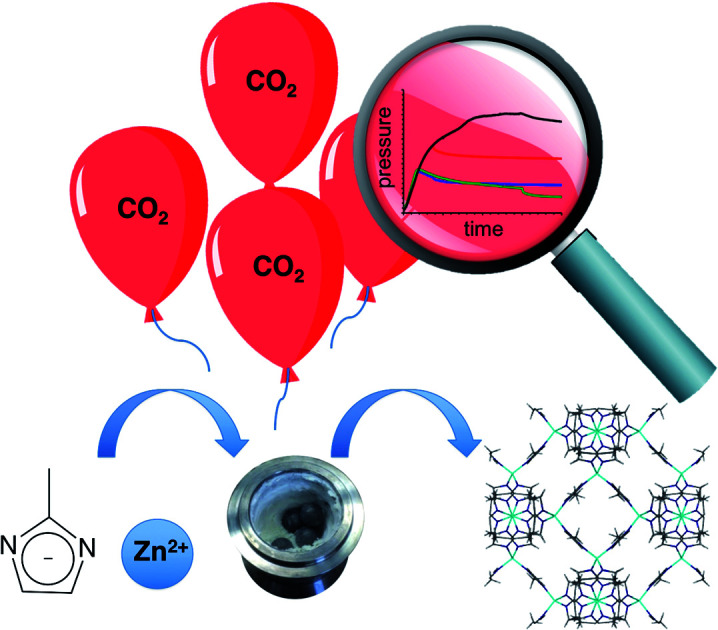

## Introduction

Mechanochemical reactions^1^ have developed from a laboratory curiosity to a viable alternative to conventional solution-based chemistry, enabling room-temperature reactions without bulk solvents,^2^ improved or previously unknown reactivity,^3^ and routes to molecules and materials otherwise difficult to access.^4^ Ball milling, twin screw extrusion,^5^ and accelerated aging^6^ have been applied successfully for the synthesis of diverse Metal–Organic Frameworks (MOFs),^7^ including carboxylate-based HKUST-1,^8^ MOF-74 ^9^ and IRMOF materials,^10,11^ zeolitic imidazolate frameworks (ZIFs),^12^ and zirconium-based UiO- and NU-systems.^13^ Importantly, mechanochemistry permits simple, room-temperature and solvent-free assembly of MOFs from metal oxides, carbonates or other basic salts: reagents that are preferred for industrial synthesis, but are often not usable due to poor solubility.^14–16^

Despite the rapid growth of applications of mechanochemistry in chemical and materials synthesis, the underlying reaction mechanisms and kinetics remain poorly understood, and it was not until 2013 that methodologies for direct, *in situ* monitoring of such transformations were reported, using powder X-ray diffraction (PXRD) and/or Raman spectroscopy.^17^ However, other simpler methods have been employed for monitoring mechanochemical reactions, in cases where direct acquisition of structural data is not required. One of these methods is monitoring the pressure and temperature of a gaseous reactant or product in a reaction vessel of constant volume – manometric monitoring. This method appears particularly attractive as the body of work on mechanochemical reactions that involve gases^18,19^ is increasing, and so is the accessibility of equipment for handling gas pressure under mechanochemical conditions. It is therefore surprising that there are comparably few reports on manometric monitoring of mechanochemical reactions, the vast majority of which involve purely inorganic systems. For example, the high-pressure mechanochemical synthesis of inorganic hydrides,^20^ nitrides,^21^ or oxides^22^ has been monitored through the uptake of H_2_, N_2_ or O_2_ gas, respectively, and the synthesis of SnO^23^ and BaWO_4_ (ref. 24) from carbonate precursors was monitored by measuring the increase in the pressure of released CO_2_. Very recently, the Borchard group used monitoring of evolved HCl gas as a way to follow the course of a mechanochemical Scholl reaction,^25^ and thermally-induced increase in gas pressure was used to detect highly exothermic reactions,^26^ demonstrating the potential for a broader applicability of manometric monitoring in studies of mechanochemistry.

Surprisingly, this approach has not yet been used to monitor mechanochemical synthesis of MOFs, despite the wide applicability and overwhelming interest for better understanding of these reactions. We hypothesized that mechanochemical synthesis of MOFs could be studied by manometric monitoring if metal carbonates were used as the metal source, releasing carbon dioxide and water. It was previously shown that metal carbonates can be used in mechanochemical synthesis of MOFs^27^ and, more broadly, coordination polymers and discrete metal–organic complexes.^28^ Basic zinc carbonate, basic copper(ii) carbonate, as well as rare earth metal carbonates have been used to synthesize diverse carboxylate-based MOFs, including pillared MOFs and HKUST-1.^27^ Moreover, the archeypal zeolitic imidazolate framework ZIF-8 has been synthesized^5^ by twin screw extrusion from 2-methylimidazole and basic zinc carbonate.

**Fig. 1:**
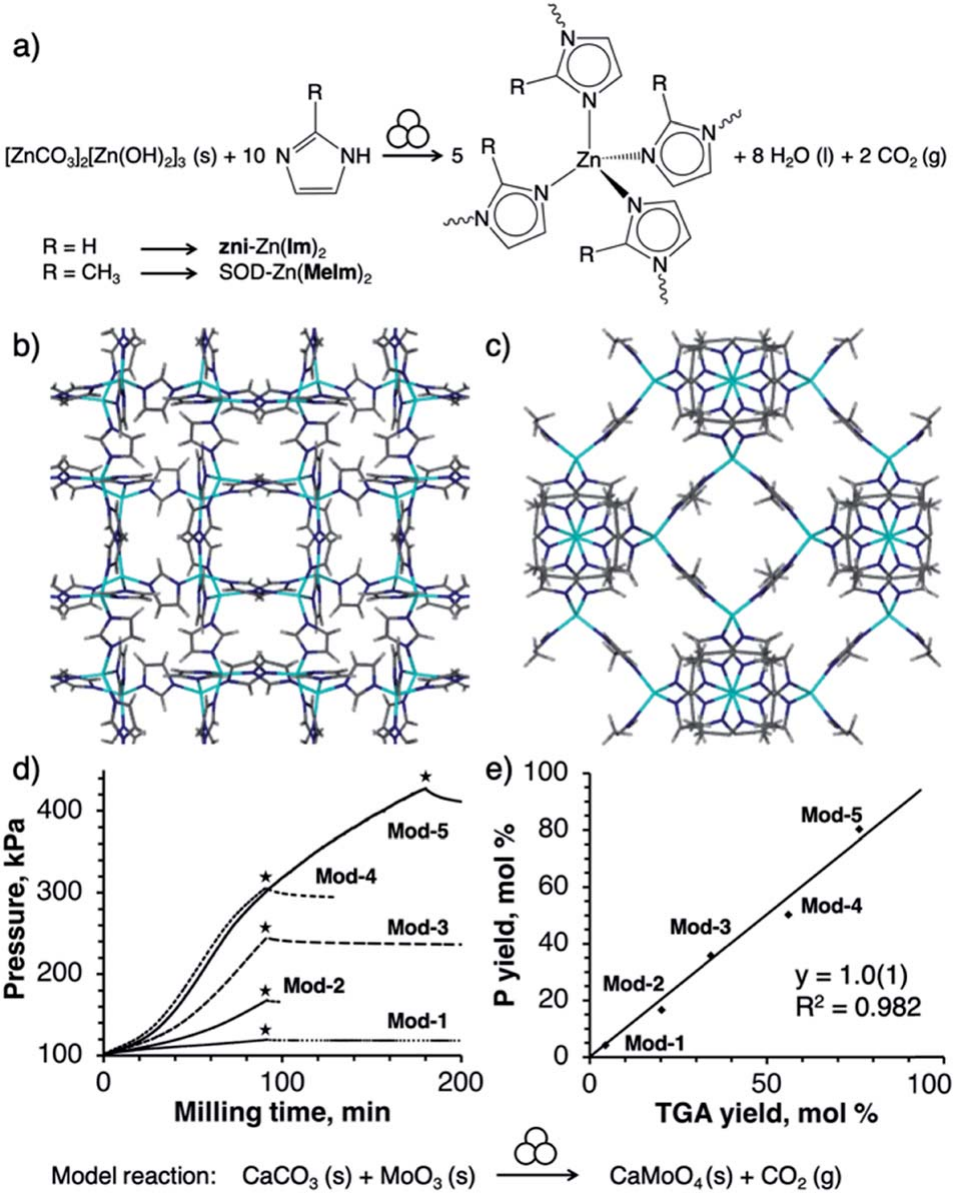
(a) Reaction scheme for mechanochemical synthesis of ZIFs from basic zinc carbonate and an imidazole. Symbol for mechanochemical conditions adopted from Righmire and Hanusa.^1*d*^ (b) Structure of **zni**-Zn(**Im**)_2_ (CSD code IMIDZB03), (c) structure of SOD-Zn(**MeIm**)_2_ (CSD code KAMZUV), (d) time-dependent pressure profiles (stars mark end of milling) and (e) correlation between pressure yield and TGA yield in the model milling reactions **Mod-(1–5)** in which solids CaCO_3_ and MoO_3_ yield solid CaMoO_4_ and CO_2_ gas (see ESI†).

Here we provide proof-of-concept of a manometric method to monitor the course of mechanochemical MOF formation by milling. Manometric monitoring allows observation of distinct differences in reactivity using three techniques: neat milling, liquid-assisted grinding (LAG)^29^ and ion- and liquid-assisted (ILAG).^30^ Specifically, we show how the use of a metal carbonate^5,27,28^ precursor can enable monitoring of the mechanochemical syntheses of MOFs, namely the **zni**-topology zinc imidazolate^31^ and the sodalite (SOD) topology zinc 2-methylimidazolate^32^ MOF (ZIF-8/MAF-4, sold as Basolite Z1200 or Porolite Z8, Fig. 1a–c). We also provide a preliminary demonstration that the methodology is applicable to an analogous cobalt(ii) system, leading to a mixture of **cag**- and **zni**-topology cobalt(ii) imidazolate (see ESI†). Importantly, we also show that manometric monitoring of reaction progress, based on changes of pressure (and temperature) inside the reaction vessel due to release of CO_2_, allowed the discovery of a feedback mechanism that creates a complex metal imidazolate carbonate side product. Identification of this mechanism enabled us to circumvent side product formation and design the first mechanochemical multi-gram synthesis of ZIF-8 without using any liquids in either the synthesis or activation step.

## Results and discussion

### Model reactions

Reactions were conducted in a Retsch PM400 planetary mill, using PM Grind Control™ vessels equipped for real-time measurement and wireless recording of pressure and temperature inside the vessel. To establish the chemical significance of pressure measurements, we first investigated a simple model reaction of solid CaCO_3_ with MoO_3_ which, upon milling in equimolar amounts, is expected to yield CaMoO_4_ and one equivalent of CO_2_ gas (Fig. 1d and e). In a typical experiment, 5.9 g of MoO_3_ (0.04 mol) and 4.1 g of CaCO_3_ (0.04 mol) were milled at a frequency of 350 rpm. Reaction conditions (milling time and media) were varied to achieve a broad range of yields (see ESI†). During milling, pressure in the reaction vessel increased in a monotonic fashion, aside from a small pressure drop at the end of the milling time associated with a rapid temperature drop. Reaction conversions were calculated both from the final pressure in the reaction vessel and from subsequent thermogravimetric analysis (TGA) of the milled samples, giving values in excellent agreement (Fig. 1e).

### Reactions of basic zinc carbonate and imidazole

**Fig. 2:**
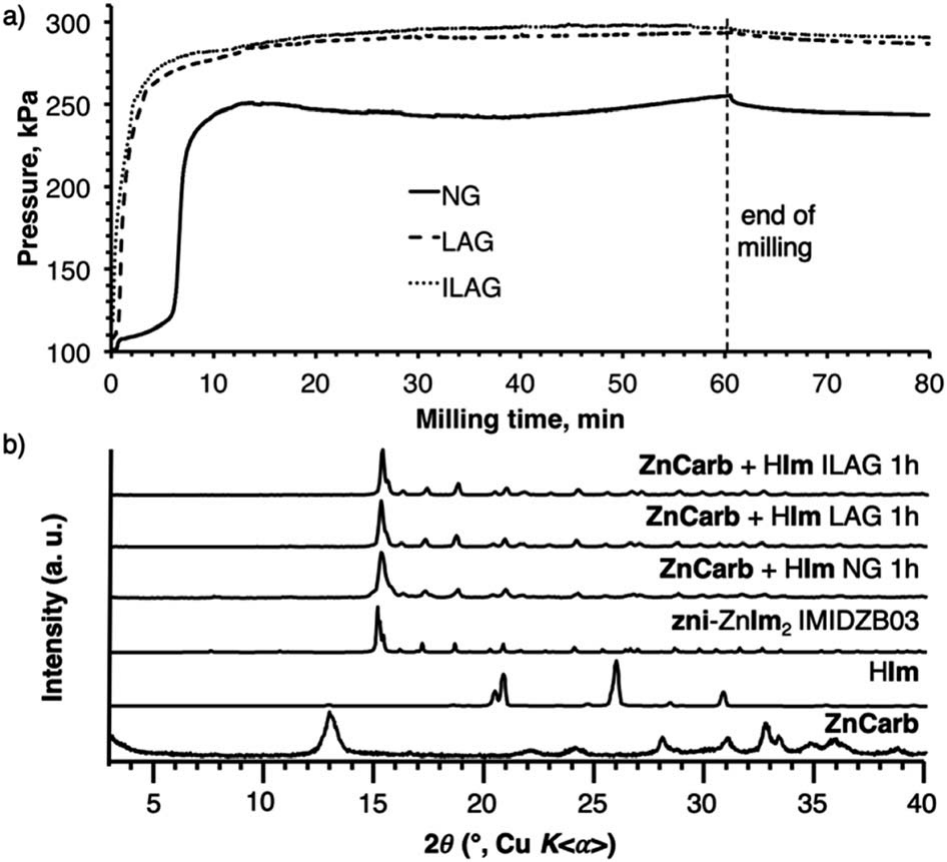
(a) Time-dependent reaction vessel pressure profiles for the mechanochemical reactions of basic zinc carbonate [ZnCO_3_]_2_[Zn(OH)_2_]_3_ with imidazole by NG (solid line), LAG (ethanol, dashed line), and ILAG (ethanol, NH_4_NO_3_, dotted line); (b) experimental PXRD patterns of the reagents and products of milling syntheses of **zni**-Zn(**Im**)_2_ from basic zinc carbonate and imidazole, and the calculated PXRD pattern of **zni**-Zn(**Im**)_2_ (CSD code IMIDZB03).

Having established that monitoring the increase of gas pressure during milling can be used to quantify the extent of reaction, we explored the neat grinding (NG) mechanochemical reaction of imidazole (H**Im**) with basic zinc carbonate, [ZnCO_3_]_2_[Zn(OH)_2_]_3_ (**ZnCarb**) in a Zn : **Im** ratio of 1 : 2. Pressure inside the vessel rose slowly during the first 5 min of milling, after which it rapidly increased, reaching a maximum within *ca.* 12 min (Fig. 2a). A significantly higher reaction rate was observed if the reaction was done by liquid-assisted grinding (LAG,^29^ milling in the presence of a liquid additive) or by ion- and liquid-assisted grinding (ILAG, milling in the presence of a liquid additive and a catalytic amount of a salt^30^). Specifically, the addition of 3 mL of ethanol (EtOH) to 13.5 g of the reaction mixture (liquid-to-solid ratio *η* = 0.22 μL mg^−1^ (ref. 29)) led to a rapid increase of pressure immediately upon the onset of milling, reaching a maximum after *ca.* 10 min (Fig. 2a). Similar observations were made for ILAG, conducted with addition of 3 mL of EtOH and 400 mg (10 mol% with respect to zinc) of NH_4_NO_3_ as the salt additive (Fig. 2a).

The LAG and ILAG reactions led to significantly higher final reaction pressures compared to neat milling, indicating conversions of 87% for LAG and ILAG and 64% for neat milling, after accounting for the vapour pressure of EtOH and solubility of CO_2_ in EtOH (see ESI†). However, analysis of the products by PXRD immediately after milling revealed that the **zni**-topology framework **zni**-Zn(**Im**)_2_ was the only crystalline component (Fig. 1b), suggesting that conversion was, in fact, complete. Moreover, TGA of the milling products, conducted after washing with ethanol, provided an excellent match for pure Zn(**Im**)_2_, without any residual zinc carbonate reactant. The TGA and PXRD results strongly indicate that all three milling approaches result in quantitative conversion of reactants to **zni**-Zn(**Im**)_2_, in contrast to reaction vessel pressure measurements. As a potential explanation for the discrepancy between pressures measured for neat milling and for reactions in the presence of a liquid phase, we consider that some of the CO_2_ gas might be retained in the pores^33^ of **zni**-Zn(**Im**)_2_ formed by neat grinding. In the case of LAG or ILAG reactions, the pores of **zni**-Zn(**Im**)_2_ might also be occupied by the liquid additive, reducing such entrapment of CO_2_.

### Reactions of basic zinc carbonate and 2-methylimidazole

#### Reactions in a 1 : 2 stoichiometric ratio of **ZnCarb** and 2-methylimidazole

**Fig. 3:**
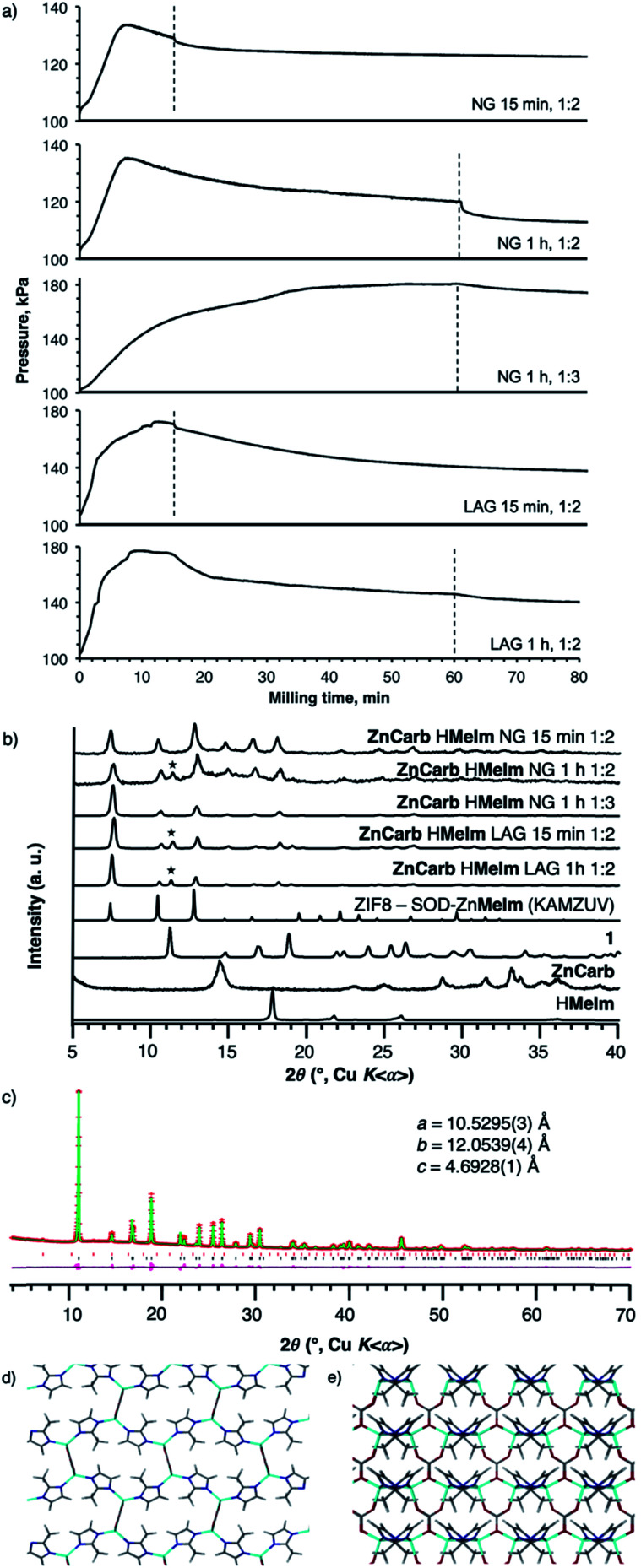
(a) Time-dependent reaction vessel pressure profiles and (b) PXRD patterns for the different syntheses of SOD-Zn(**MeIm**)_2_ from basic zinc carbonate and H**MeIm**. Top to bottom: NG reactions; Zn : H**MeIm** = 1 : 2 (15 min and 1 h), Zn : H**MeIm** = 1 : 3 (1 h), ethanol LAG reactions; Zn : H**MeIm** = 1 : 2 (15 min and 1 h). Stars indicate peaks of **1** in the reaction mixtures. Structure determination of complex carbonate **1** from PXRD data: (c) the final fit obtained in the Rietveld refinement, and views of the structure of **1** along (d) the *c*-axis and (e) the *a*-axis.

Next, we investigated reaction vessel pressure changes for the reaction of basic zinc carbonate and 2-methylimidazole (H**MeIm**), expected to yield the popular sodalite (SOD) topological form of Zn(**MeIm**)_2_ (ZIF-8). The reaction vessel pressure profile was very different from that observed with H**Im**. Milling the two reactants in a 1 : 2 stoichiometric ratio of **ZnCarb** and H**MeIm** (15 g scale), led to a monotonic increase in vessel pressure over the first 10 min milling, followed by a slow decrease (Fig. 3a). The appearance of such a temporary peak in reaction vessel pressure was observed in all repeated experiments. PXRD analysis of the reaction products after 15 min milling revealed only Bragg reflections of ZIF-8. However, after 1 h milling, PXRD analysis of the products revealed additional peaks, consistent with a complex metal carbonate (**1**) of unknown structure, previously reported^34^ to form upon exposure of ZIF-8 to moist CO_2_. Close examination of the ^1^H → ^13^C cross-polarization magic angle spinning (CP-MAS) solid-state nuclear magnetic resonance (SSNMR) spectrum of the product after 15 min milling shows signals of **1**, indicating that it is present in the mixture, though undetected by PXRD (see Fig. S14†). Similar behavior was observed with LAG, conducted by adding 3 mL of EtOH to 15 g of reaction mixture (*η* = 0.20 μL mg^−1^). In this case, the reaction vessel pressure also exhibited an early maximum, and slowly diminished over time. Analysis by PXRD again revealed the formation of ZIF-8, along with low-intensity reflections of **1**.

These observations suggest that the mechanochemical reaction of basic zinc carbonate and H**MeIm** proceeds in two stages, first yielding ZIF-8 and producing an increase in the reaction vessel pressure due to CO_2_ evolution. Further milling, however, leads to adsorption of the nascent CO_2_ gas by ZIF-8 and further reaction between ZIF-8 and the H_2_O and CO_2_ byproducts, which yields the carbonate phase **1** and is accompanied by a decrease in the reaction vessel pressure. This proposed reaction pathway was also verified by analogous experiments in a shaker mill, the smaller scale of which enabled more thorough sampling of the reaction mixture. Analysis of PXRD patterns of the LAG reaction mixture after 5 min and 10 min milling revealed that ZIF-8 was the only reaction product, while Bragg reflections of **1** were clearly visible after 30 min (Fig. S21†). Such reaction behavior was not greatly affected by the choice of milling liquid (water, methanol or isopropanol), although it seems that the use of sterically more demanding milling liquids leads to less carbonate byproduct (Fig. S22†).

#### Crystal structure determination of **1** from PXRD data

In order to substantiate that a carbonate phase is indeed formed on reaction of ZIF-8 and moist CO_2_, crystal structure determination of **1** was carried out directly from PXRD data^35^ recorded on a sample prepared (independently) by exposing a suspension of ZIF-8 in water to a stream of CO_2_ gas. The PXRD data were recorded in transmission mode on a Bruker D8 instrument (Ge-monochromated CuK_α1_), and revealed that the sample contained a small residual amount of ZIF-8 together with **1**. The PXRD data for **1** were indexed using the ITO code^36^ in the program CRYSFIRE,^37^ giving a unit cell with orthorhombic metric symmetry (*a* = 10.54 Å, *b* = 12.06 Å, *c* = 4.70 Å; *V* = 596.5 Å^3^). The space group was assigned as *Pba*2, with two formula units of Zn_2_(**MeIm**)_2_CO_3_ in the unit cell (*Z* = 2). Profile fitting using the Le Bail method in the GSAS program^38^ gave a good quality of fit (*R*_wp_ = 3.99%, *R*_p_ = 2.81%; Fig. S25†), and the refined unit cell parameters were used in the subsequent structure solution calculation, which was carried out using the direct-space genetic algorithm technique in the program EAGER.^39^ The best structure solution was then used as the starting model for Rietveld refinement, carried out using the GSAS program,^38^ giving a good final Rietveld fit (*R*_wp_ = 4.97%, *R*_p_ = 3.37%; Fig. 3c) to the PXRD data, very close to the quality of the Le Bail fit, with the following refined parameters: *a* = 10.5295(3) Å, *b* = 12.0539(4) Å, *c* = 4.6928(1) Å, *V* = 595.62(4) Å^3^.

In **1**, each zinc cation is bonded tetrahedrally to two **MeIm−** and two CO_3_^2−^ anions. The 2-methylimidazolate anions bridge the zinc cations into one-dimensional ribbons, which are then connected into sheets by carbonate anions (Fig. 3d). The CO_3_^2−^ anions display two modes of coordination: one of the oxygen atoms is doubly coordinated and connects zinc atoms within a single sheet, while the other two oxygen atoms are singly coordinated to two neighbouring zinc atoms in an adjacent sheet (Fig. 3e). In this way, the carbonate serves both as a building block for the sheets and as a perpendicular strut stacking the sheets together into a three-dimensional framework.

#### Reactions in a 1 : 3 stoichiometric ratio of **ZnCarb** and H**MeIm**

The manometric observation of the reaction between basic zinc carbonate and H**MeIm** in the respective 1 : 2 stoichiometric ratio (discussed above) revealed a parasitic process that converts the target ZIF-8 into a complex carbonate **1**. The composition of **1** suggests that the process could involve a reaction in which **MeIm−** anions are replaced by carbonates:

1 2Zn(**MeIm**)_2_ + H_2_O + CO_2_ ⇌ Zn(**MeIm**)_2_·ZnCO_3_ + 2H**MeIm**

The proposed equation suggests that formation of **1** may be avoided by using excess H**MeIm**, both as a pore-filling agent to minimize access of CO_3_^2−^ to zinc centers, and as a means to take advantage of Le Chatelier's principle to shift the equilibrium towards the formation of ZIF-8.

Manometric monitoring of the neat milling reaction of **ZnCarb** with three equivalents of H**MeIm** per zinc revealed a continuous increase in reaction vessel pressure, which plateaued after *ca.* 50 min milling (Fig. 3a and S13†). PXRD analysis of the sample after 1 h milling revealed ZIF-8 as the only reaction product, with no observable Bragg reflections due to **1** (Fig. S12†). After washing with EtOH, TGA of the product gave a residue of 36% (Fig. S18†), which is consistent with the ZnO residue expected for pure ZIF-8 (36.4%). Furthermore, the ^1^H → ^13^C CP-MAS SSNMR spectrum of the product after 15 min milling showed only ZIF-8 peaks, and no signals due to **1** (Fig. S20†). Similarly, PXRD analysis of analogous LAG reactions with excess H**MeIm** in a shaker mill showed no evidence for the presence of **1** even after 30 min milling with methanol, ethanol or isopropanol as the liquid additives (Fig. S23†). Interestingly, none of the milling products from the Zn : H**MeIm** = 1 : 3 preparations showed evidence of H**MeIm** in the PXRD pattern, in line with the hypothesis that extra ligand is encapsulated in the pores of ZIF-8.

### Solventless synthesis and purification of 90 g of ZIF-8

We have shown that using excess H**MeIm** in the mechanochemical reaction of basic zinc carbonate and H**MeIm** facilitates the formation of large quantities of pure ZIF-8 at room temperature under mild conditions. However, the fact that washing with solvent is still required as part of the purification process detracts somewhat from the environmental benefits of using mechanochemistry. Consequently, a fully solvent-free method for synthesizing and purifying/activating MOFs would be desirable. To tackle this problem, we prepared ZIF-8 mechanochemically from basic zinc carbonate at a 90 g scale and purified it without using solvents, providing the very first demonstration of a MOF synthesized without using any solvents at any stage of preparation or activation. Specifically, the product resulting from a neat grinding reaction of basic zinc carbonate and H**MeIm** (Zn : H**MeIm** = 1 : 3) carried out in a planetary mill was purified by sublimation (200 °C under vacuum for 5 h) of the included H**MeIm** without any detrimental effect on the ZIF-8, as corroborated by PXRD, TGA and N_2_ sorption isotherms (Fig. S25–S27†). In addition, this route yielded a MOF with 1785 m^2^ g^−1^ BET surface area, comparable to the commercial analogue (1758 m^2^ g^−1^). The sublimed H**MeIm** was collected as colourless crystals and was found to be pure by solution-state ^1^H NMR spectroscopy, allowing the possibility of reusing the excess H**MeIm**.

### Scope of the methodology

In principle, the carbonate-based synthesis and reaction monitoring methodology presented herein should be applicable to a wide range of MOF materials. Indeed, the use of metal carbonate reactants was already investigated^27^ by Yuan *et al.* for zinc-based pillared MOFs and rare earth-based carboxylate MOFs, while Riccò *et al.* reported the synthesis of HKUST-1 from basic copper(ii) carbonate. As a preliminary exploration of the wider applicability of this synthetic methodology, we also explored the reaction of cobalt(ii) carbonate (CoCO_3_) with H**Im** in 1 : 2 molar ratio, both by neat milling and by LAG with methanol. The results (see ESI†) overall show slower reaction kinetics than in the analogous reaction using basic zinc carbonate, but again demonstrate significantly faster reactivity in the case of LAG; thus, after 90 min milling, pressure-based measurements indicate a reaction conversion of 42% for neat milling and 92% for LAG. Analysis of the products by PXRD after 90 min milling indicates qualitatively different behavior compared to the zinc-based system: neat milling leads to a predominantly amorphous material, while LAG produces a mixture of products, including **zni**-Co(**Im**)_2_ and the less dense **cag**-Co(**Im**)_2_ framework. More detailed studies on this system and analogous 2-methylimidazolate derivatives (*e.g.* ZIF-67) are ongoing.

## Conclusions

In summary, we have shown for the first time that ZIFs (namely **zni**-Zn(**Im**)_2_ and SOD-Zn(**MeIm**)_2_) can be made mechanochemically from basic zinc carbonate, without any solvent use at all, and that the reaction progress can be monitored *in situ* by measuring the change of pressure inside the milling vessel due to CO_2_ released in the reaction. Such manometric monitoring of the synthesis of the commercially viable ZIF-8 revealed an unexpected feedback loop, in which the newly produced ZIF-8 reacts with released CO_2_ gas and water to form a complex zinc carbonate methylimidazolate, the structure of which has been elucidated here, and provides a new addition to the already rich landscape of zinc 2-methylimidazolate phases.^40^ Formation of the byproduct was then prevented by addition of excess ligand, enabling large-scale quantitative synthesis of ZIF-8 from inexpensive and available precursors, and the first example of a totally solvent-free route for the mechanochemical manufacture and activation of this MOF.

## Materials and methods

Details of experimental procedures are given in ESI.† Large scale (*ca.* 15 g) mechanochemical syntheses were carried out in a Retsch PM400 mill operating at 300 rpm. Reaction mixtures were milled in a 250 mL PM Grind Control™ reaction vessel with seven stainless steel balls (*m* = 44 g, *d* = 20 mm, *V* = 6 mL). Small scale (200 mg) reactions were done in a 10 mL stainless steel milling jar with two stainless steel balls (*m* = 1.3 g, *d* = 7 mm) at 30 Hz. All samples were washed extensively with ethanol to remove potential unreacted imidazole starting material before further analysis. The vessel pressure curves, TGA, PXRD, solid-state NMR and FTIR-ATR data are given in the ESI,† along with details of PXRD structure determination of **1**. Crystallographic data for **1** have been deposited with the Cambridge Structural Database (CCDC deposition code 1942361).

## Conflicts of interest

There are no conflicts to declare.

## Supplementary Material

SC-011-C9SC05514B-s001

SC-011-C9SC05514B-s002
