# Supplementary material for: Manometric real-time studies of the mechanochemical synthesis of zeolitic imidazolate frameworks
Source: Chem Sci. 2020 Jan 2;11(8):2141–7. doi: 10.1039/c9sc05514b (PMC8150112; doi:10.1039/c9sc05514b)
Supplement: SC-011-C9SC05514B-s001 [file SC-011-C9SC05514B-s001.pdf]

Electronic Supplementary Material (ESI) for Chemical Science.
This journal is © The Royal Society of Chemistry 2020

Manometric real-time studies of the mechanochemical synthesis of zeolitic imidazolate frameworks

- Supplementary Information-

Ivana Brekalo, Wenbing Yuan, Cristina Mottillo, Yuneng Lu, Yuancheng Zhang, Jose Casaban, K. Travis Holman*, Stuart L. James*, Frédéric Duarte, P. Andrew Williams, Kenneth D. M. Harris* and Tomislav Friščić*

Abstract:

We demonstrate a simple method for real-time monitoring of mechanochemical synthesis of metal-organic frameworks, by measuring changes in pressure of gas produced in the reaction. Using this manometric method to monitor the mechanosynthesis of the zeolitic imidazolate framework ZIF-8 from basic zinc carbonate reveals an intriguing feedback mechanism in which the initially formed ZIF-8 reacts with the CO_2 byproduct to produce a complex metal carbonate phase, the structure of which is determined directly from powder X-ray diffraction data. We also show that the formation of the carbonate phase may be prevented by using excess ligand, enabling the rational development of first design leading to completely solvent-free and quantitative mechanochemical formation of ZIF-8 on a 90 gram scale, with excess ligand removed by sublimation.

Table of Contents

1. Experimental Procedures 2
   1.1. General Details 2
   1.2. Instrumental Details 2
2. Results and Discussion 4
   2.1. Pressure yield calculation 4
   2.2. Model reaction of molybdenum(VI) oxide and calcium carbonate 5
   2.3. Basic zinc carbonate and imidazole experiments 9
      2.3.1. Large-scale milling reaction with temperature and pressure monitoring 9
      2.3.2. Small-scale milling reaction 13
   2.4. Basic zinc carbonate and 2-methylimidazole experiments 15
      2.4.1. Large-scale milling reaction with temperature and pressure monitoring 15
         2.4.1.2. Liquid assisted grinding experiments 18
      2.4.2. Small-scale experiments 23
   2.5. Large scale solvent-free synthesis and purification of SOD-Zn(MeIm)$_2$ 28
   2.6. Structure determination of compound 1, $Zn_2(MeIm)_2CO_3$, directly from PXRD data 30
   2.7. Cobalt (II) carbonate and imidazole experiments 31
3. References 33
4. Author Contributions 33

1. Experimental Procedures

1.1. General Details

Basic zinc carbonate (>58%, Zn basis), imidazole (98%), and 2-methylimidazole (99%), were purchased from Sigma Aldrich. Ammonium nitrate (Certified ACS) was purchased from Fisher. Molybdenum (VI) oxide (99.5%) was purchased from Alfa Aesar. Calcium carbonate (99.5%) was purchased from BDH Chemicals. Ethanol (95%) was purchased from Commercial Alcohols. Methanol and isopropanol were purchased from ACP. All chemicals were used without further purification.

1.2. Instrumental Details

Time-dependent pressure and temperature profiles in large-scale milling reactions were collected using 250 mL PM GrindControl™ jars supplied by Retsch GmbH. The milling was performed in a Retsch PM 400 planetary mill operating at 300-350 rpm, with the addition of either 4-7 medium-size steel balls (m ≈ 32 g, V = 4 mL, d = 17 mm) in the model reaction experiments, or with 7 large steel balls (m ≈ 44 g, V = 6 mL, d = 20 mm) in ZIF synthesis experiments. To ensure the jars were sealed gas-tight, vacuum-grade silicon grease was used on the seal, and the jar ring clamps were wrench-tightened. To avoid cross-contamination, the milling balls and jars were cleaned by milling a mixture of sodium carbonate and laboratory solid detergent (Sparkleen) with added ethanol for 15 min after every use. Analysis of data was conducted using the PM GrindControl™ software and Microsoft Excel.

Small scale milling reactions were conducted in a 10 mL stainless steel jar with one 7 mm (1.4 g) and one 9 mm (3.5 g) stainless steel ball. The samples were milled at 30 Hz for 5-30 min using a Retsch MM400 ball mill.

Powder X-ray diffraction (PXRD) patterns were collected using a Bruker D2 powder diffractometer equipped with a Cu-Kα (λ=1.54060 Å) source and Lynxeye detector set at a discriminant range of 0.110 V to 0.250 V. The patterns were collected in the range of 3° to 40°. Analysis of PXRD patterns was conducted using Panalytical X'Pert Highscore Plus software. Experimental patterns were compared to simulated patterns calculated from published crystal structures using Mercury crystal structure viewing software. Crystallographic Information Files containing published crystal structures were obtained from the Cambridge Structural Database (CSD) and Crystallography Open Database (COD).
High-quality PXRD data for use in structure determination calculations were recorded on a Bruker D8 instrument using Ge-monochromated $CuK\alpha_1$ radiation. The powder XRD data were recorded in transmission mode (2θ range, 2 – 70°; step size, 0.017°; total data collection time, 16 h 51 m) with the sample held between two pieces of tape (i.e., foil-type sample holder).

Fourier-transform infrared attenuated total reflection (FTIR-ATR) spectra were collected using a Bruker Vertex 70 FTIR-ATR spectrometer in the range 400 cm-1 to 4000 cm-1. FTIR spectra were analysed using Bruker OPUS software and Microsoft Excel.

Thermogravimetric analysis (TGA) analyses were conducted on a Mettler Toledo TGA/DSC 1 STARe System. All samples were heated at a rate of 5°C/min from 25°C to 800°C under dynamic atmosphere of air with a flow rate of 60 mL/min. The flow rate of the protective gas (N2) was 40 mL/min. TGA curves were analysed using Mettler Toledo TGA analysis software.

Solid-state ^{13}C CP-MAS NMR spectra were collected on a 400 MHz Varian VNMR equipped with a 7.5 mm CPMAS probe at a spin rate of 5 KHz. All spectra were collected with a contact time of 2 ms and recycle delay of 2 s.

2. Results and Discussion

2.1. Pressure yield calculation

To calculate reaction yield from the pressure measurements, the difference in pressures, Δp was measured, along with the temperature at the start (T_{start}) and end of milling (T_{end}). The starting pressure was corrected to the final temperature using the Gay-Lussac law:

$$p_{start,corr} = p_{start} * (T_{end}/T_{start}) \qquad (1)$$

The difference in pressure was then used to calculate the number of moles of CO_2 produced in the reaction (n) using the ideal gas law:

$$\Delta pV = nRT \qquad (2)$$

where Δp is the corrected pressure difference between the start and end of milling at the end-of-milling temperature, V is the empty volume of the reaction vessel, R is the gas constant (8.314 J/Kmol), and T is the temperature at the end of milling. Use of the more exact Van der Waals gas law was attempted instead of the ideal gas law, but the difference in result was negligible for the range of temperatures and pressures used (less than 0.5 % difference in all cases).

The volume of the reactants (V_{react}) was estimated from their density and included in the calculation of the empty volume of vessel (V) along with the volume taken up by milling balls. The volume of the vessel alone was measured to be 288 mL, based on the volume of water it can accommodate.

$$V = 288 \text{ mL} - V_{react} - V_{ball} * \#_{\text{balls}} \qquad (3)$$

In later ZIF synthesis experiments, the vapor pressures of water[1] (formed in the reaction), and ethanol[2] (added in LAG experiments) were calculated for the given temperatures using the Antoine equation, and then subtracted from the corresponding total pressures before the rest of the calculations were performed. In addition, the amount of CO_2 gas dissolved in 3 mL of ethanol was calculated using Henry's law[3] and subtracted from the theoretical number of moles of produced CO_2 to get an accurate yield.

2.2. Model reaction of molybdenum (vi) oxide and calcium carbonate

To independently validate reaction yields obtained by pressure measurements, we took as a model reaction the solid state reaction of calcium carbonate and molybdenum(VI) oxide which releases CO_2 gas according to the following equation:

$$CaCO_3\ (s) + MoO_3\ (s) \rightarrow CaMoO_4\ (s) + CO_2\ (g)$$

In a typical reaction, 5.9 g of MoO_3 (0.04 mol) and 4.1 g of $CaCO_3$ (0.04 mol) were milled in a 250 mL steel jar with 4-7 medium-size balls, using a PM 400 planetary mill at a frequency of 350 rpm for 90-270 min. The number of balls and milling time were varied in order to obtain a range of yields adequate for building a calibration curve. The pressure and temperature in the jars were measured during milling, and the products were analyzed *via* PXRD and TGA after standing in a desiccator for 1-2 h to remove any adsorbed CO_2.

An example of the real-time graphical output for the model reaction **Mod-4** is shown in Figure S1. The temperature of the gas inside the vessel rises during milling, more rapidly at first, then slower as milling goes on. At end of milling the temperature drops rapidly, then continues to slowly fall off. The pressure rises in a quasi-sigmoidal fashion, possibly indicating an induction period, followed by reaction progress, then as more of the reagents are spent, the reaction slows down. At end of milling, the pressure rapidly falls, following the fall in temperature of the gas inside the vessel. Near identical temperature behavior is seen in all experiments (model reactions, as well as later ZIF syntheses), but the pressure behavior can change drastically depending on the system in question.

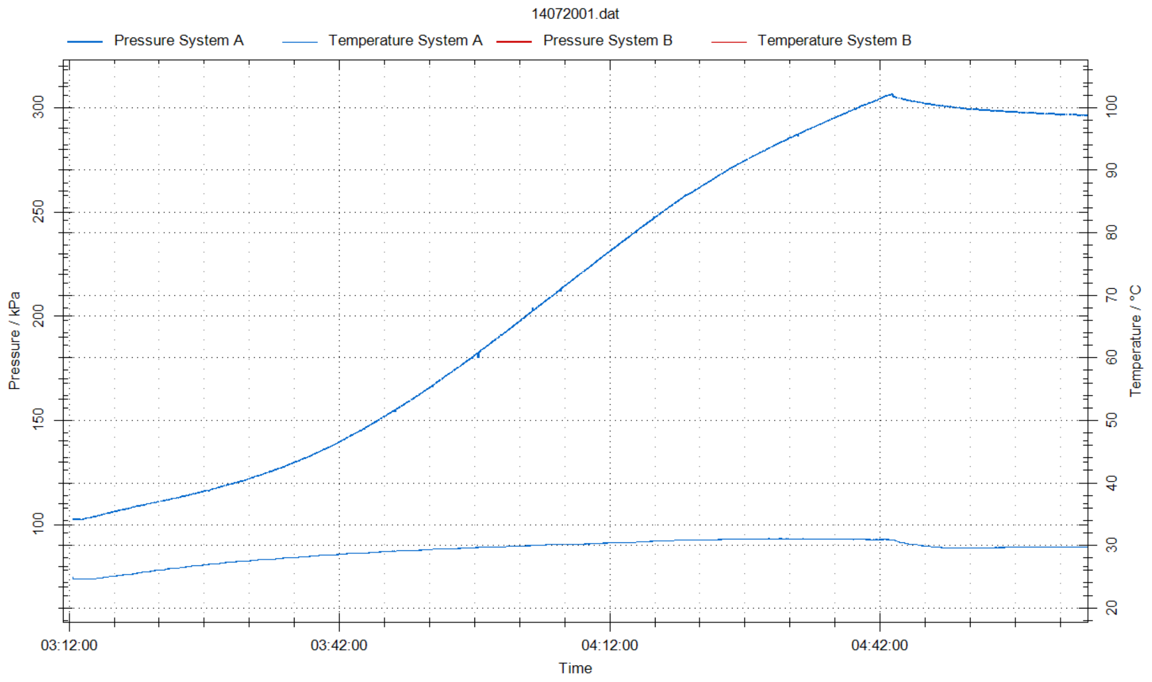

Figure S1. Time-dependent pressure (top line) and temperature (bottom line) profiles of model milling reaction **Mod-4** between $CaCO_3$ and MoO_3 (90 min milling with 7 balls).

Vessel pressure yield was calculated for all model reactions according to section S2.1. The yield was also calculated from TGA analysis by measuring the overall mass loss between room temperature and 650 °C (Step 1 and 2, see example TGA curve for **Mod-3** in Figure S5). As both MoO_3 and $CaMoO_4$ decompose above 650 °C, any mass loss below that temperature corresponds to CO_2 release; either due to thermally induced reaction of leftover reagents, or due to calcium carbonate decomposition, and can be used to calculate the number of moles of CO_2 released thermally, and thus the amount of unreacted $CaCO_3$.

Table S1 shows the range of reaction conditions and corresponding pressure and TGA yields, while the pressure and temperature profiles, and the model reaction curves are shown in Figure S2. The pressure and TGA yields show very good agreement, with a linear plot, R^2 = 0.9821 (Figure S3).
Figure S4 shows PXRD patterns of samples **Mod-1 - Mod-5**. Interestingly, the yield cannot be accurately determined from PXRD data, most likely due to amorphization of the reagents.

Table S1. Experimental data and TGA and pressure yield for model reaction experiments

Sample code	**Mod-1**	**Mod-2**	**Mod-3**	**Mod-4**	**Mod-5**
Milling time / min	90	90	90	90	180
Number of balls	4	5	6	7	7
Pressure yield / %	4.4	16.9	36.0	50.6	80.6
TGA yield / %	4.2	20.0	33.9	55.8	75.7

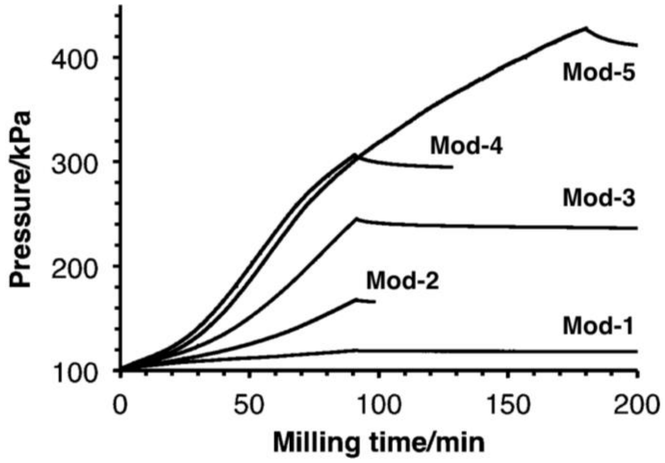

Figure S2. Time-dependent pressure profiles of model milling reactions **Mod-(1-5)** between $CaCO_3$ and MoO_3.

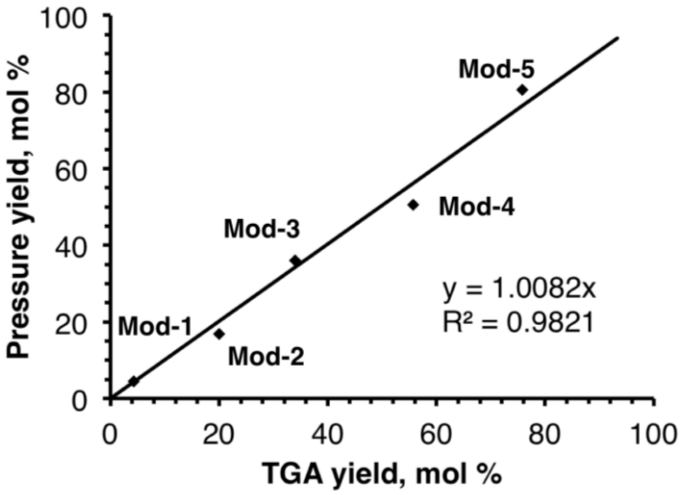

Figure S3. Dependence of pressure yield on TGA yield in the model milling reactions **Mod-(1-5)** between $CaCO_3$ and MoO_3.

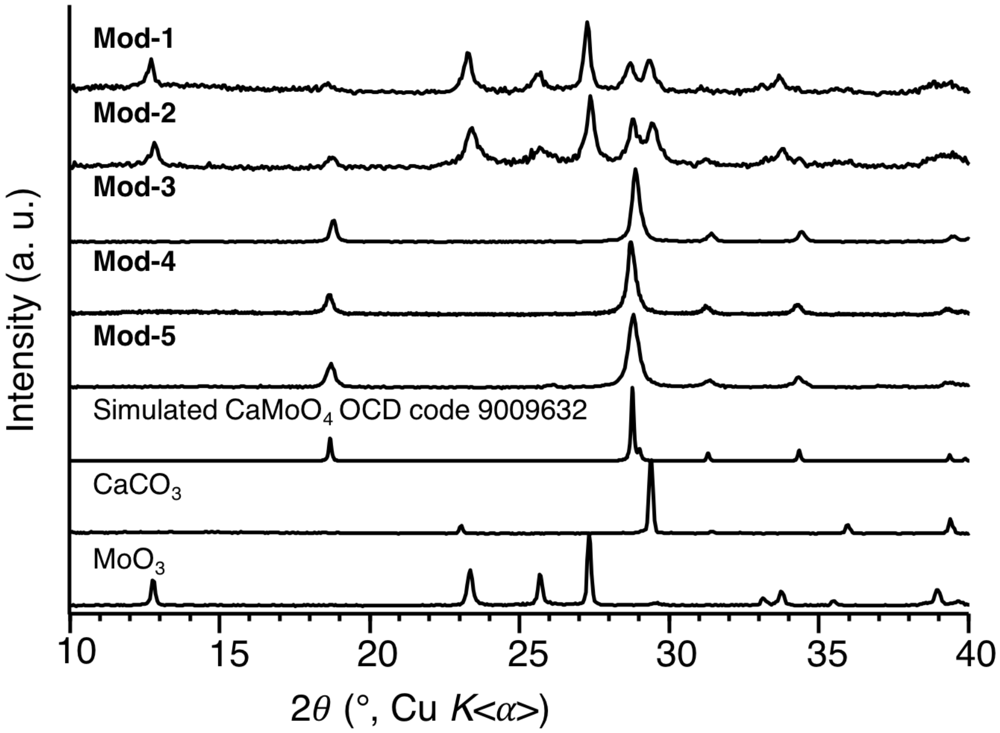

Figure S4. Comparison of experimental PXRD patterns of the starting reagents and the products of model milling reactions **Mod-(1-5)** between $CaCO_3$ and MoO_3, as well as the simulated PXRD pattern of pure $CaMoO_4$.

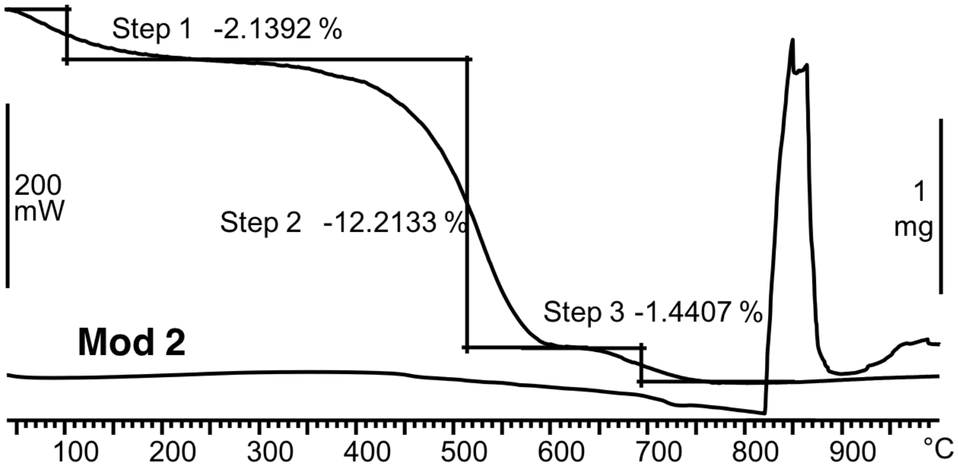

Figure S5. Representative TGA curve in a dynamic atmosphere of air for the product of the model reaction between $CaCO_3$ and MoO_3, **Mod-2**.

2.3. Basic zinc carbonate and imidazole experiments

We then explored the solid state reaction of basic zinc carbonate (**ZnCarb**) and imidazole (H**Im**) which releases CO_2 gas according to the following equation:

$$[ZnCO_3]_2[Zn(OH)_2]_3\,(s) + 10H\mathbf{Im}(s) \rightarrow 5\ Zn\mathbf{Im}_2(s) + 8\ H_2O(l) + 2\ CO_2(g)$$

2.3.1. Large-scale milling reaction with temperature and pressure monitoring

In a typical large-scale reaction, 6.0 g of basic zinc carbonate (0.01 mol, 0.05 mol of Zn) and 7.5 g of imidazole (0.1 mol) were milled in a 250 mL steel jar with 7 large balls, using a PM 400 planetary mill at a frequency of 300 rpm for 1 h. For liquid assisted grinding (LAG) experiments 3 mL of ethanol were added to the reaction mixture, and for the ion and liquid assisted (ILAG) experiments 3 mL of ethanol and 0.4 g (0.005 mol, or 10 mol% based on Zn) of ammonium nitrate were added. The pressure and temperature in the jars were measured during milling (example of representative data is shown in Fig S7). The milled samples were stirred in 50 mL of ethanol for approx. 15 min, vacuum filtered, and then washed with an additional 50 mL of ethanol to ensure removal of unreacted imidazole, and dried on vacuum for 15 min. The washed samples were analyzed *via* PXRD (Fig S6) and TGA (Fig S9) after standing in a desiccator for 1-2 h. Additional experiments were conducted without immediate washing of products, to confirm that the conversion is not due to the washing procedure.

All syntheses resulted in the formation of pure **zni**-Zn**Im**$_2$ as the only product (Figure S6). There were no signs of remaining reagents in any of the cases, indicating that the conversion based on PXRD is 100%. The products before and after washing are also identical based on PXRD, as shown in Figure S6, meaning that washing with ethanol doesn't change the reaction outcome, purely removes potential excess imidazole.

The NG synthesis reaction vessel pressure showed, similar to the model reactions, a quasi-sigmoidal curve with an induction period, a quick rise in product formation, and then a tapering off of the reaction progress as the reagents were spent (Figure S7, solid line). Surprisingly, the reaction vessel pressure appeared to reach its maximum after only 15 minutes! To ensure reproducibility, several measurements were repeated, and are shown to be in good correspondence (Figure S8). Conversely, the LAG and ILAG syntheses (Figure S7, dashed and dotted lines, respectively) showed almost no induction period, and a quasi-exponential growth, reaching near-maximum pressure after less than 10 minutes. The maximum pressures (and calculated conversions) of the LAG and ILAG reactions were very similar and higher than that of the NG reaction.

Interestingly, the yields as calculated by TGA (Figure S9) are higher than the vessel pressure yields, as seen in Table 2. We hypothesize that this is due to absorption of CO_2 by the newly formed ZIF. This is in line with the fact that pressure yields of LAG and ILAG preparations are significantly higher than that of the NG synthesis, possibly due to the additives blocking access to the product voids and preventing CO_2 absorption.

Table 2. Comparison of TGA and vessel pressure yields for the large-scale reaction of basic zinc carbonate and imidazole.

	NG	LAG	ILAG
TGA yield/%	99.4	98.4	99.8
pressure yield/%	64.0	86.8	87.2

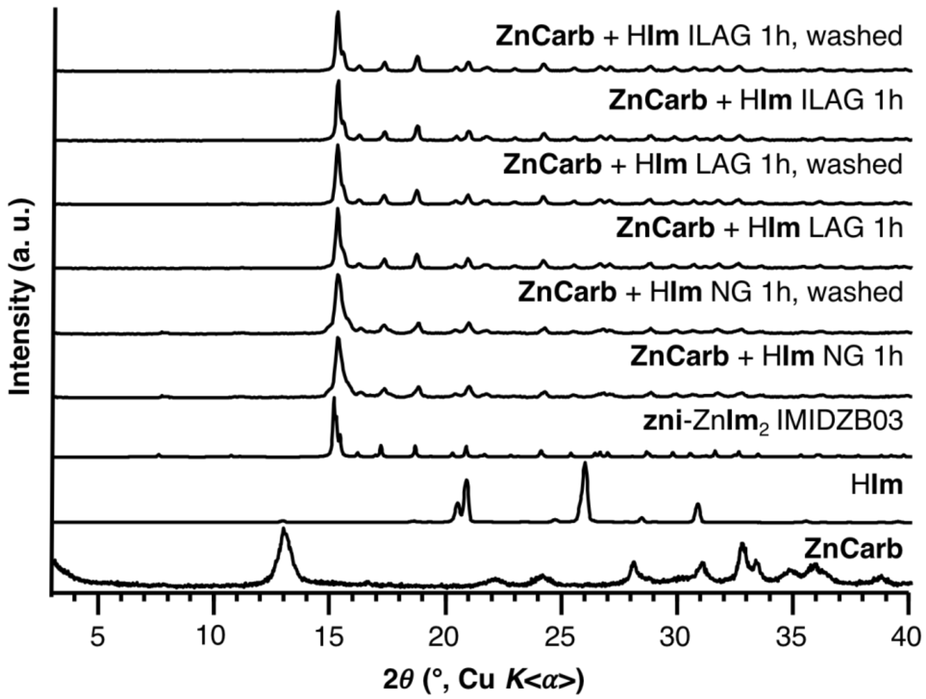

Figure S6. Comparison of the PXRD patterns for the products of the large-scale milling reactions (reaction time was 1 hour in all cases) of basic zinc carbonate and imidazole using different mechanochemical methods (NG, LAG, ILAG), washed, and unwashed, as well as the PXRD patterns of the starting reagents.

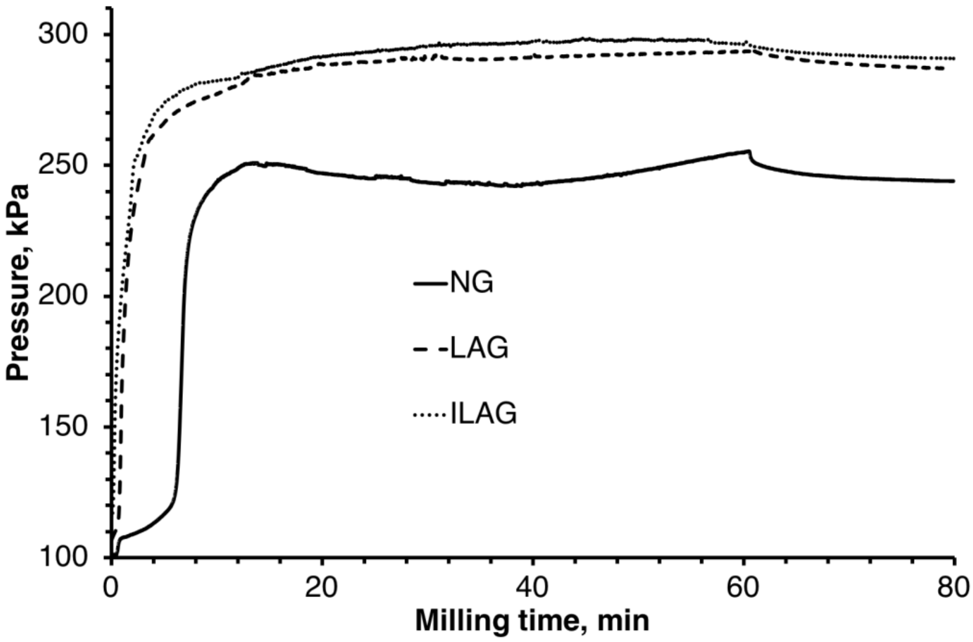

Figure S7. Gas pressure measurements for the neat grinding (NG, solid), liquid assisted grinding (LAG, dashed) and ion-and-liquid assisted grinding (ILAG, dotted) reactions of basic zinc carbonate and imidazole. The milling liquid for LAG and ILAG is ethanol, and the salt additive for ILAG is NH_4NO_3, and reaction time was 1 hour in all cases.

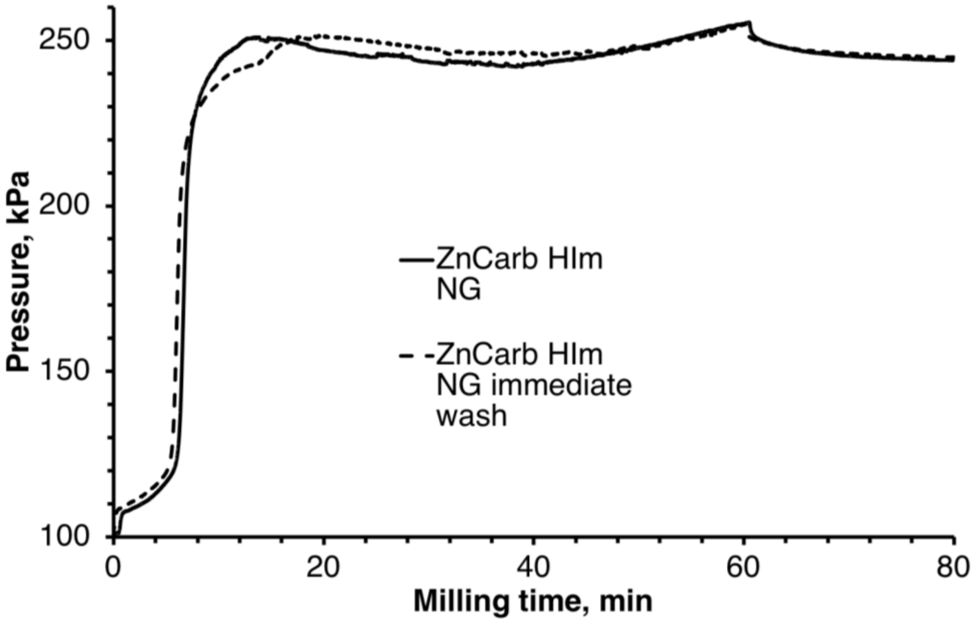

Figure S8. Reproducing gas pressure measurements for the 1h NG reaction of basic zinc carbonate and imidazole, with a milling time of 1 hour.

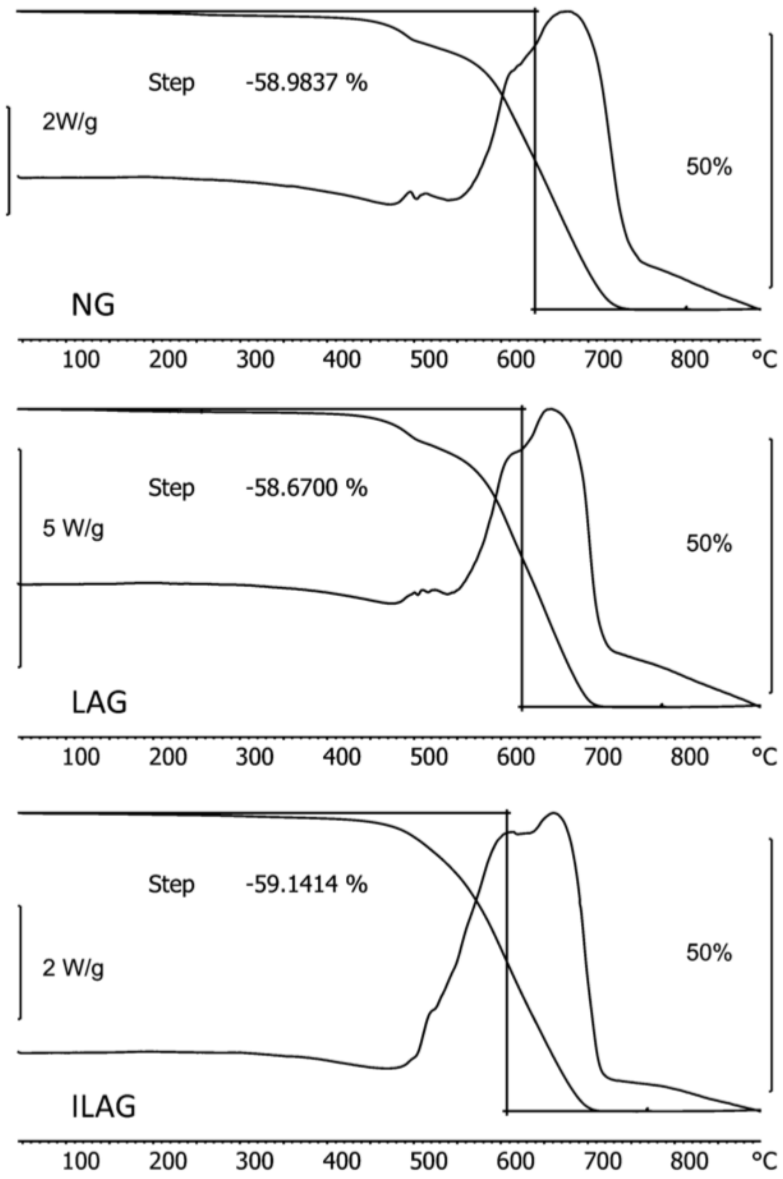

Figure S9. Comparison of TGA curves (measured in a dynamic atmosphere of air) of the washed products of different large-scale milling reactions between basic zinc carbonate and imidazole (NG, LAG, and ILAG). In all cases the milling time was 1 hour.

2.3.2. Small-scale milling reaction

In a typical small-scale reaction, 92.0 mg of basic zinc carbonate (0.168 mmol, 0.838 mmol of Zn) and 114.1 mg of imidazole (1.68 mmol), were milled in a 10 mL PMMA milling jar with one 7 mm diameter (1.3 gram), and one 10 mm diameter (3.5 g) stainless steel ball at 30 Hz for 30 min. For liquid assisted grinding (LAG) experiments 75 μL of ethanol were added to the reaction mixture, while for ion-and-liquid assisted grinding (ILAG) experiments, an additional 6.5 mg of ammonium nitrate (10 mol% based on Zn) was added. PXRD and FT-IR measurements were performed without washing the samples (Figure S10 and S11, respectively).

Similar to large-scale experiments, the PXRD patterns of all products show full conversion to **zni**-Zn**Im**$_2$. The FT-IR of the LAG and ILAG reactions shows the presence of ethanol, consistent with our hypothesis that it is adsorbed onto the product, potentially blocking access to CO_2 molecules entering the product voids, and resulting in a smaller difference between the TGA and pressure yield than that in NG experiments.

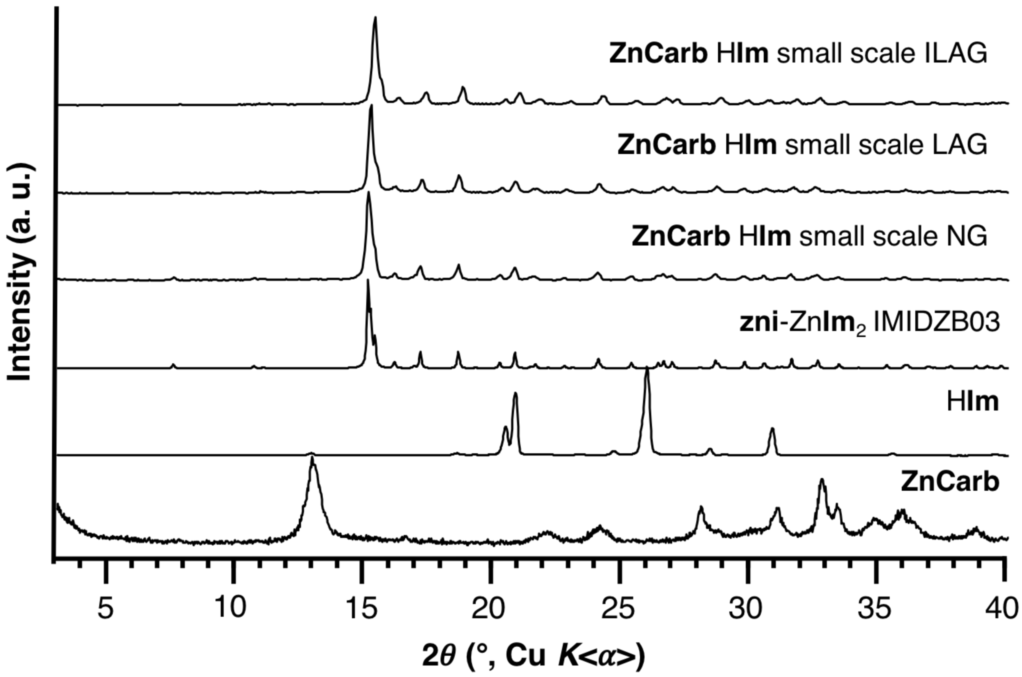

Figure S10. Overlay of PXRD patterns for the unwashed products of small scale 30 min milling reactions of basic zinc carbonate and imidazole under different conditions (NG, LAG, ILAG). In all cases the milling time was 30 minutes.

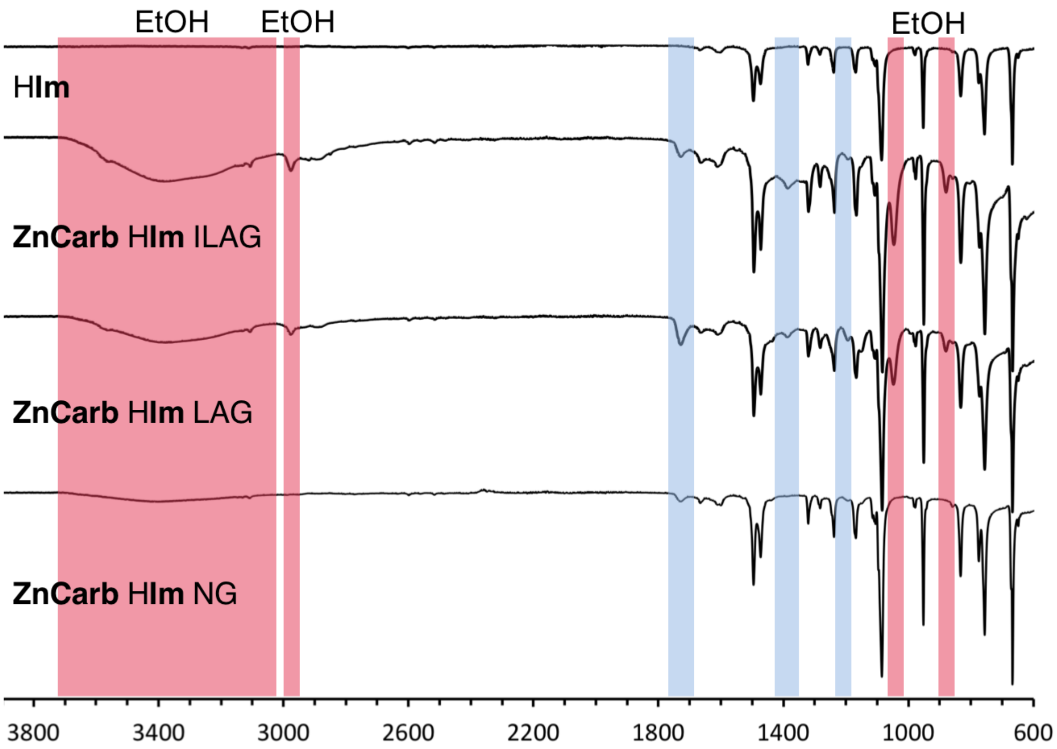

Figure S11. Comparison of FT-IR spectra of small scale syntheses of **zni**-Zn**Im**$_2$ by milling basic zinc carbonate and imidazole (top to bottom: H**Im**, products of the ILAG, LAG, and NG synthesis).

2.4. Basic zinc carbonate and 2-methylimidazole experiments

We further explored the solid state reaction of basic zinc carbonate and 2-methylimidazole (H**MeIm**) which releases CO_2 gas according to the following equation:

$$[ZnCO_3]_2[Zn(OH)_2]_3\,(s) + 10H\mathbf{MeIm}(s) \rightarrow 5\,Zn\mathbf{MeIm}_2(s) + 8\,H_2O(l) + 2\,CO_2(g)$$

Reactions with H**MeIm** were performed with two different reactant ratios, Zn:H**MeIm** = 1:2 or 1:3, and on a small (200 mg), as well as large scale (15 g).

2.4.1. Large-scale milling reaction with temperature and pressure monitoring

In a typical Zn:H**MeIm** = 1:2 large scale reaction, 6.0 g of basic zinc carbonate (0.01 mol, 0.05 mol of Zn) and 9.0 g of 2-methylimidazole (0.1 mol) were milled in a 250 mL steel jar with 7 large balls, using a PM 400 planetary mill at a frequency of 300 rpm for 15 min and (in separate experiments) for 1 h. In a Zn:H**MeIm** = 1:3 large-scale reaction, 4.6 g of basic zinc carbonate (0.008 mol, 0.04 mol of Zn) and 10.3 g of 2-methylimidazole (0.12 mol) were used. For liquid assisted grinding (LAG) experiments 3 mL of ethanol were added to the reaction mixture. The pressure and temperature in the jars were measured during milling. The milled samples were stirred in 50 mL of ethanol for approx. 15 min, vacuum filtered, and then washed with an additional 50 mL of ethanol to ensure removal of unreacted 2-methylimidazole, and dried on vacuum for 15 min. The washed samples were analyzed *via* PXRD (Fig S11) and TGA after standing in a desiccator for 1-2 h.

Additional experiments were conducted without immediate washing of products, to confirm that the conversion is not due to the washing procedure. PXRD and SSNMR data was collected for the products of these syntheses immediately after opening the milling jars.

2.4.1.1. Neat grinding experiments

The vessel gas pressure curves of neat grinding experiments in a Zn:H**MeIm** ratio of 1:2 (Figure S13) showed a short induction period followed by rapid pressure growth, similar to that in H**Im** milling experiments. Unlike the previous H**Im** milling experiments, however, this rapid increase was followed by a steady decline in vessel gas pressure, evening out only after milling stops. The PXRD analysis of the 1:2 ratio large-scale reaction product after 15 min shows the formation of SOD-Zn$\mathbf{Im}_2$ product (ZIF8, Figure S12). Closer inspection of the *washed* 15 min 1:2 ratio synthesis product shows a small peak at $\approx 11°2\theta$, corresponding to a previously reported complex zinc carbonate methylimidazolate, which is formed by exposing ZIF8 to wet carbon dioxide. The zinc methylimidazolium carbonate byproduct is formed in very small amounts after 15 min, so it is visible in PXRD only after washing away the amorphous background, but CP-MAS SSNMR without washing clearly shows its presence (Figure S20). The same peak can be seen in the PXRDs of both the washed, and unwashed 1:2 large-scale 1h milling product, in a much larger amount, showing that as the reaction proceeds, more of the byproduct is formed (Figure S12). This formation of byproduct is mirrored by a drop in vessel pressure (Figure S13), indicating absorption of CO_2. We therefore hypothesize that the different behavior of

vessel pressure (compared to H**Im** reactions) is due to absorption of newly formed CO_2 by the porous ZIF8 product, and subsequent formation of the unwanted complex carbonate byproduct **1**.

It follows that if the absorption of CO_2 could be prevented, the formation of carbonate could also be stopped. To that end, an additional equivalent of 2-methylimidazole was added into the milling reaction, in hope that it would help block the pores of newly formed ZIF8. PXRD of the products of this reaction (Figure S12) showed only the formation of ZIF8, with no complex carbonate byproduct peaks. The vessel pressure shape during milling was much more similar to the original H**Im** reaction (Figure S7), with a starting induction period, followed by a steady rise in pressure (albeit much slower than with the H**Im** reaction). Despite this similarity in shape, the pressure yields obtained were severely underestimated compared to the TGA yields (45% pressure yield vs. 99% TGA yield for the 1h NG reaction in a 1:3 ratio), much more so than in the H**Im** reactions. We hypothesize that some absorption of CO_2 happens even when the pores are partially blocked, but not enough to facilitate formation of the byproduct.

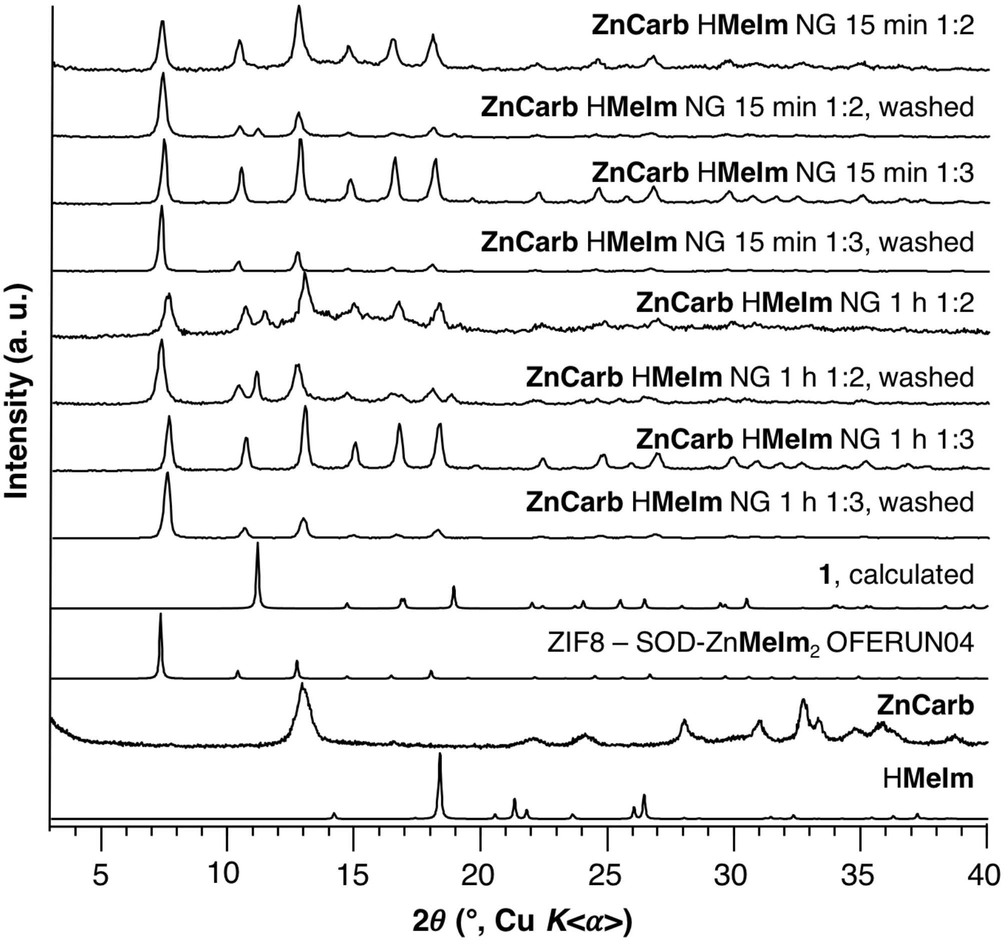

Figure S12. Comparison of PXRD patterns for the products of large-scale neat grinding reactions of basic zinc carbonate (**ZnCarb**) and 2-methylimidazole (H**MeIm**) in a Zn:H**MeIm** ratio of 1:2 or 1:3, after 15 minutes or 1 hour. The PXRD patterns of all samples are shown both before and after washing with ethanol. The calculated PXRD patterns of SOD-Zn**MeIm**$_2$ (ZIF8) and zinc 2-methylimidazolium carbonate (**1**) are given for comparison.

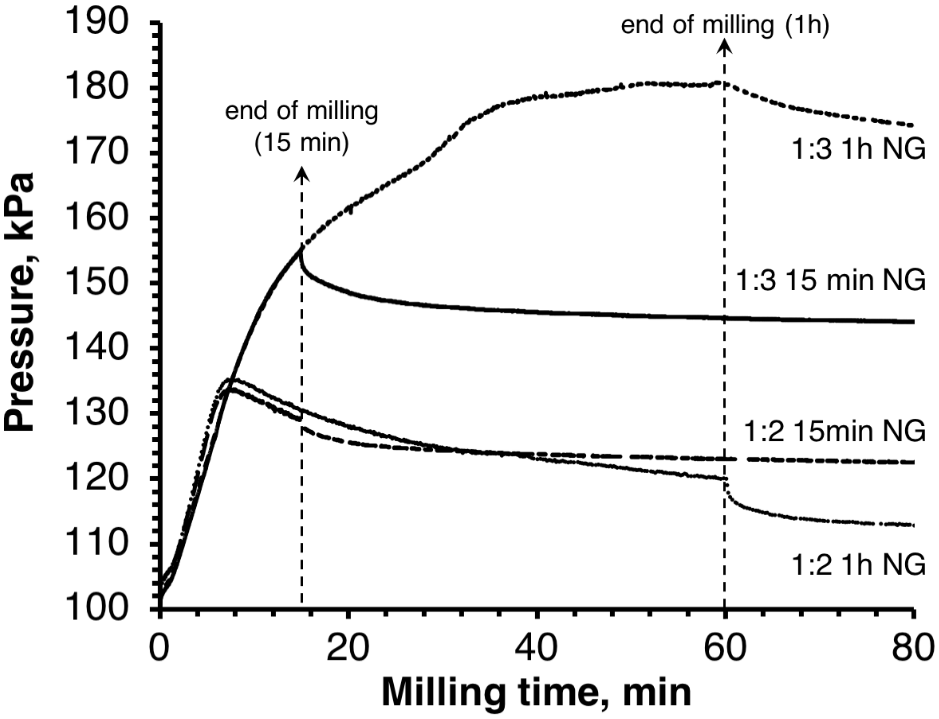

Figure S13. Gas pressure measurements for the neat grinding (NG) reactions of basic zinc carbonate and 2-methylimidazole in a zinc to H**MeIm** stoichiometric ratio of either 1:2 (1 hour grinding – long dashed line, 15 minutes grinding – short dashed line) or 1:3 (1 hour grinding – dotted line, 15 minutes grinding – solid line).

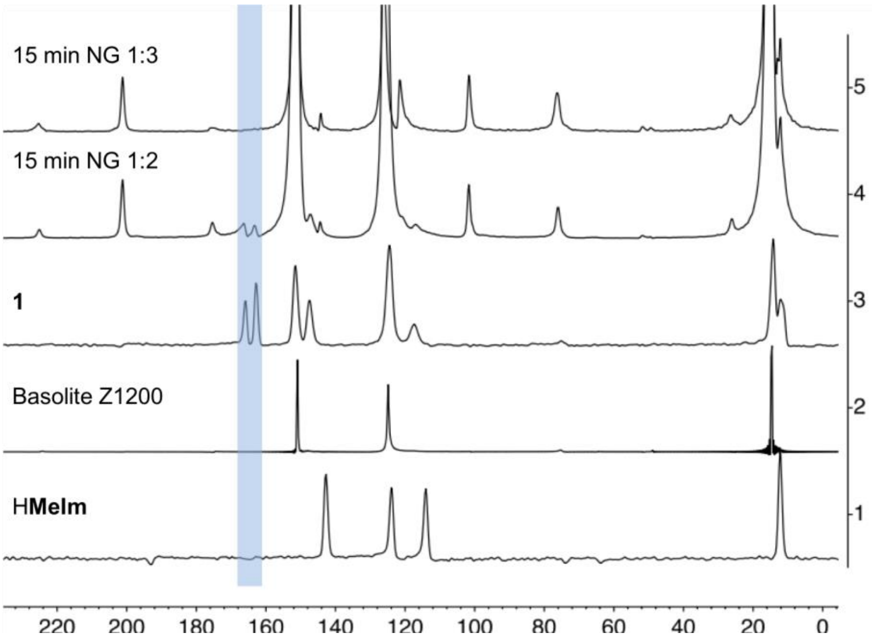

Figure S14. Comparison of CP-MAS SSNMR spectra for the products of large-scale neat milling reactions of basic zinc carbonate and H**MeIm** in respective stoichiometric ratios of 1:2 and 1:3. Milling was performed for 15 minutes in each case, and the spectra are compared to those of SOD-Zn$\mathbf{MeIm}_2$ (ZIF8, or Basolite Z1200), and of the complex zinc carbonate methylimidazolate **1**.

2.4.1.2. Liquid assisted grinding experiments

The LAG milling experiments' vessel pressure profiles (Figure S16) follow a similar trend to the NG experiments, in that 1:2 reactions show a drop in pressure after the initial rise, and show peaks of carbonate byproduct **1** in the PXRD pattern of the products after 15, and 30 minutes (both washed and unwashed, Figure S15). The LAG experiments reach an overall higher pressure before the pressure drop (Figure S17), which could be ascribed to the dual effect of the ZIF8 product being formed faster in the LAG reaction, as well as pore-filling of ZIF8 by solvent molecules, which slows down the byproduct formation. Raising the Zn:H**MeIm** ratio to 1:3 results in a vessel pressure profile that has no drops in pressure, as well as pure ZIF 8 products, based on PXRD. Expectedly, the maximum reaction vessel pressure in the LAG experiments is reached sooner than in the NG experiments, after 10-15 min. In these cases as well, the yield is greatly underestimated compared to the TGA yield.

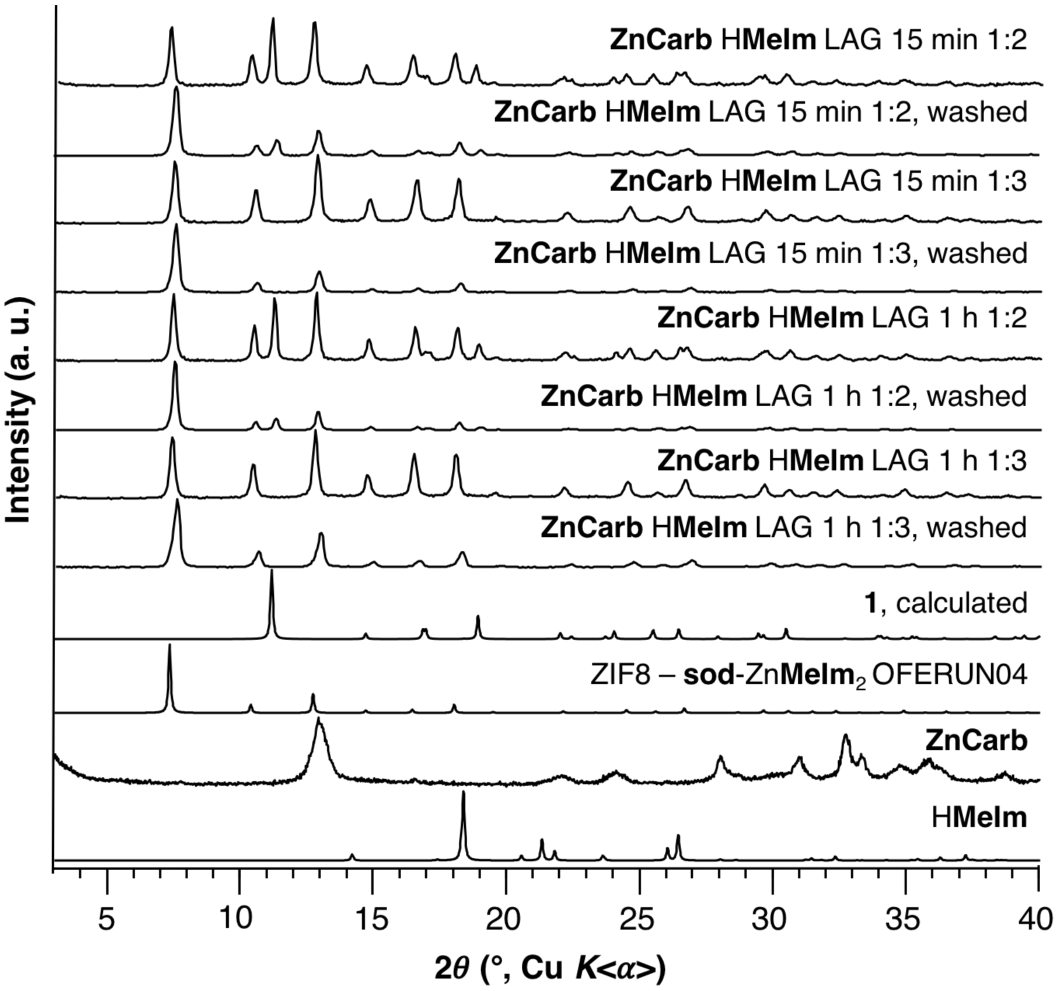

Figure S15. Comparison of PXRD patterns for the products of large-scale liquid assisted grinding reactions of basic zinc carbonate (**ZnCarb**) and 2-methylimidazole (H**MeIm**) in a respective stoichiometric ratio of zinc to H**MeIm** of 1:2 or 1:3, after 15 minutes or 1hour, with ethanol as the added liquid. The PXRD patterns of all samples are shown both before and after washing with ethanol. The calculated PXRD patterns of SOD-Zn**MeIm**$_2$ (ZIF8) and zinc 2-methylimidazolium carbonate (**1**) are given for comparison.

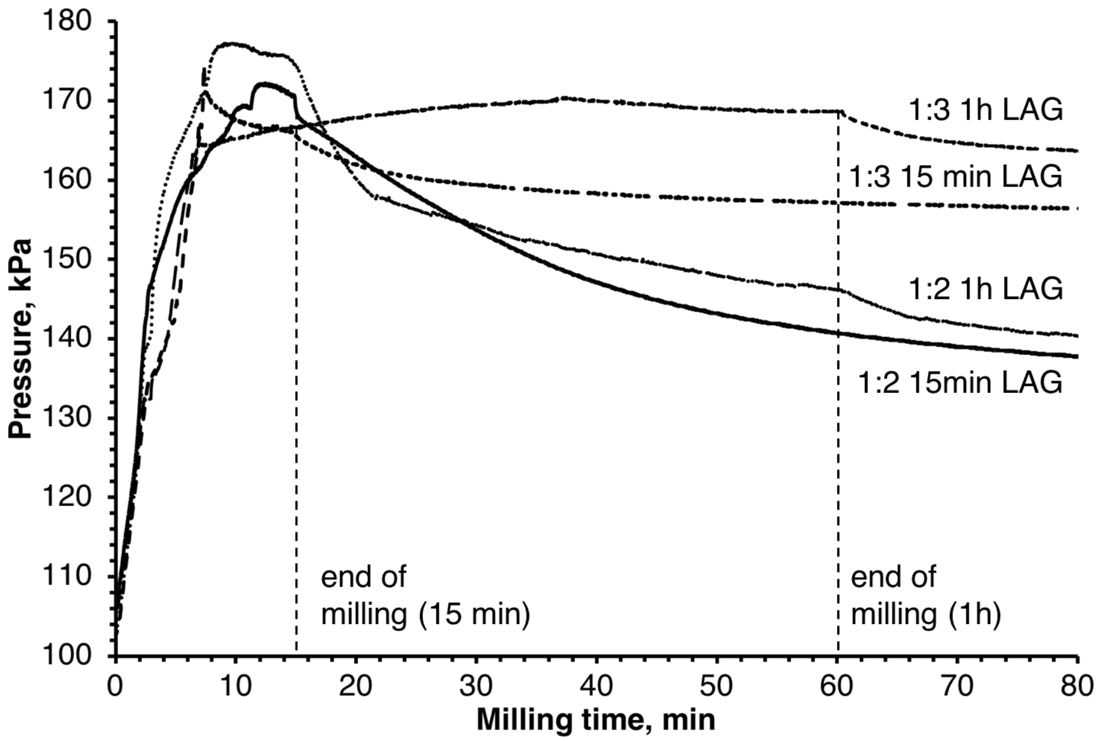

Figure S16. Comparison of gas pressure measurements for the LAG reactions of basic zinc carbonate and 2-methylimidazole in a respective stoichiometric ratio of zinc to H**MeIm** of 1:2 (1 hour grinding – dotted line, 15 min grinding – solid line) and 1:3 (1 hour grinding – large dashes, 15 minutes grinding – small dashes). In all cases ethanol is the liquid additive.

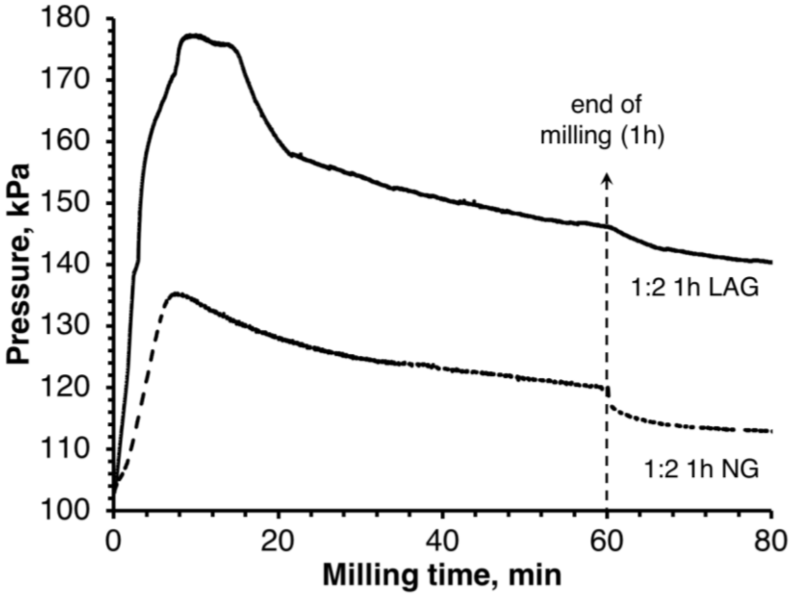

Figure S17. Gas pressure measurements for the 1 hour neat grinding (dashed line) and LAG (solid line) reactions of basic zinc carbonate and 2-methylimidazole, using a respective stoichiometric ratio of zinc to H**MeIm** of 1:2.

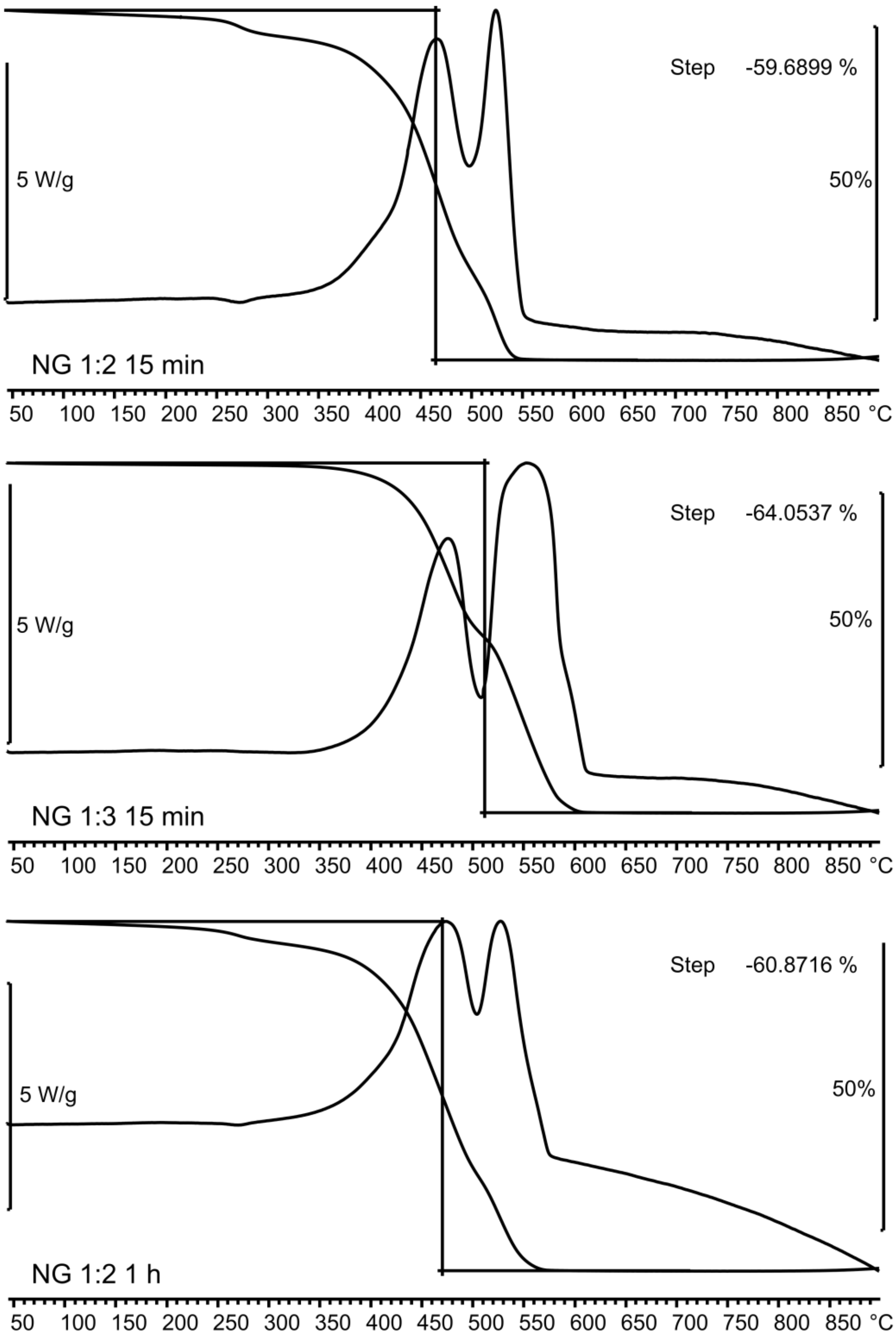

Figure S18. Comparison of TGA curves (measured in a dynamic air atmosphere) of the products of large-scale neat milling of basic zinc carbonate and H**MeIm** for 15 minutes (1:2 and 1:3 stoichiometric ratio of Zn to H**MeIm**), and 1 hour (1:2 stoichiometric ratio of Zn to H**MeIm**).

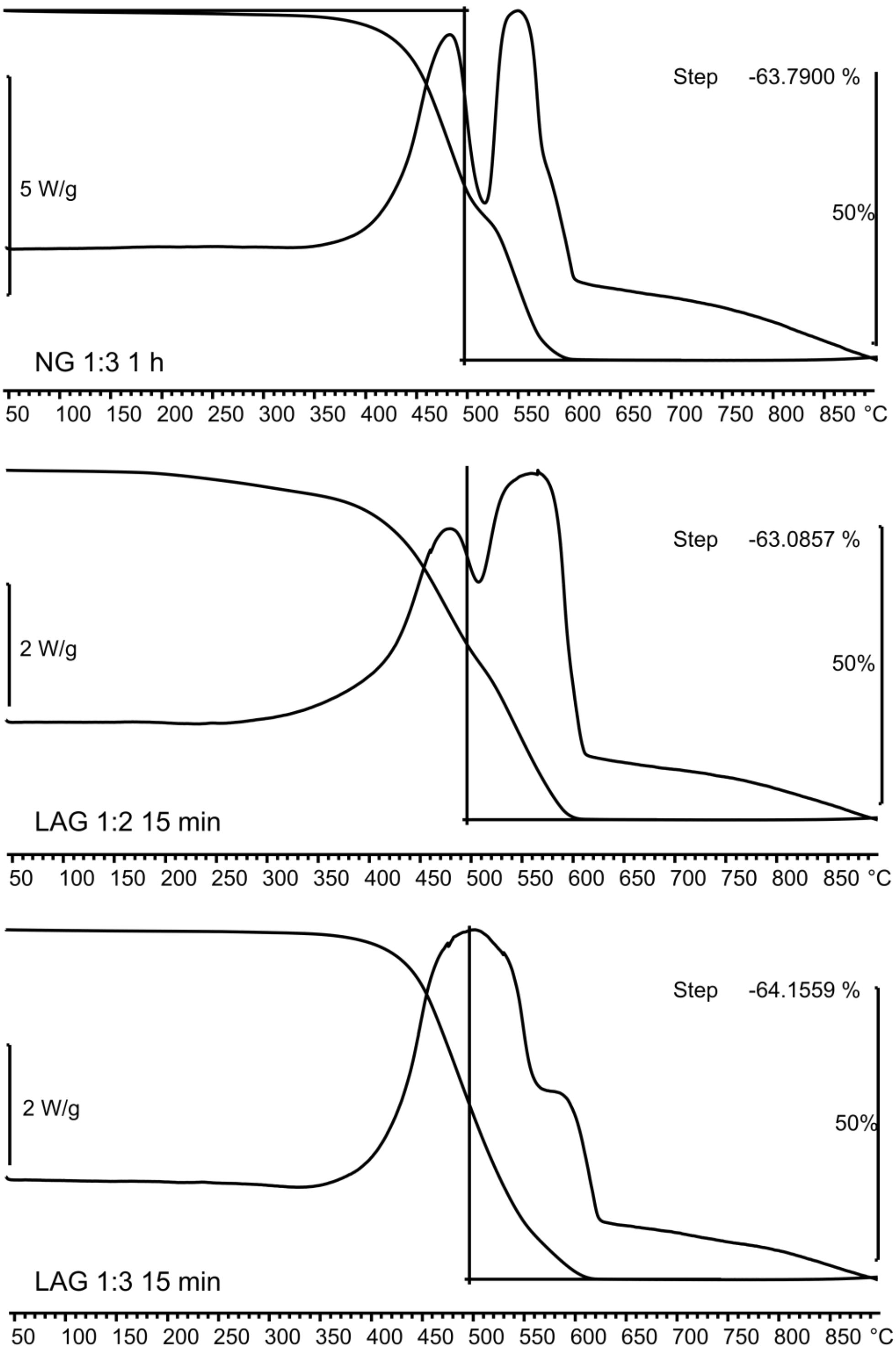

Figure S19. Comparison of TGA curves (measured in a dynamic air atmosphere) of the products of large-scale neat milling of basic zinc carbonate and H**MeIm** in a 1:3 respective stoichiometric ratio for 1 hour ratio, and large-scale LAG for 15 minutes in either a 1:2 and 1:3 respective stoichiometric ratio.

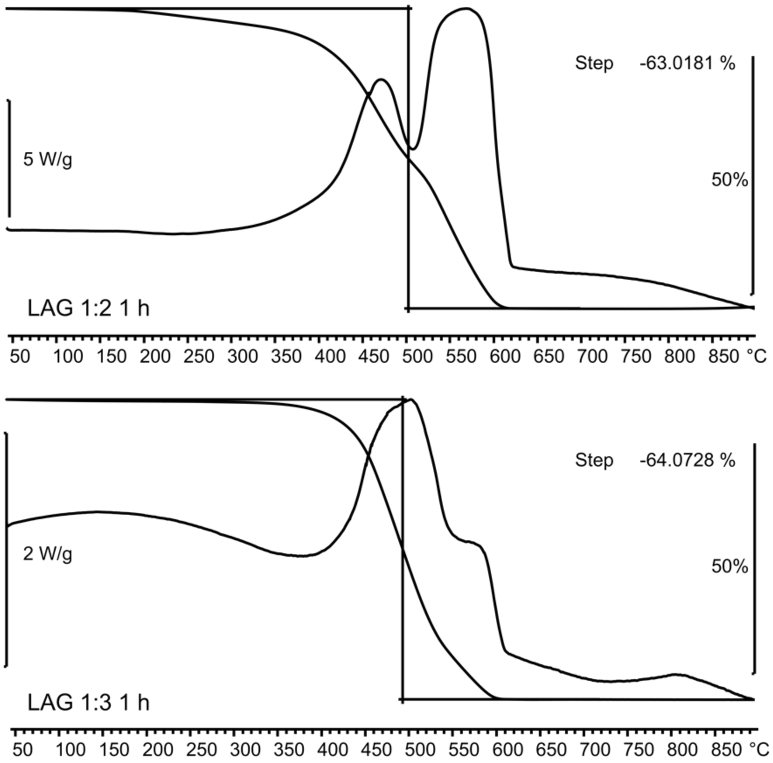

Figure S20. Comparison of TGA curves (measured in a dynamic air atmosphere) of the products of large-scale LAG reaction of basic zinc carbonate and H**MeIm** after 1 hour milling in a 1:2 and 1:3 respective stoichiometric ratios.

2.4.2. Small-scale experiments

In a typical Zn:H**MeIm** = 1:2 small scale reaction, 80.0 mg of basic zinc carbonate (0.15 mmol, 0.75 mmol of Zn) and 119.5 mg of 2-methylimidazole (1.5 mmol) were milled in a 10 mL PMMA milling jar with one 7 mm diameter (1.3 gram), and one 10 mm diameter (3.5 g) stainless steel ball at 30 Hz for 10, 15, or 30 min (in separate experiments). For the Zn:H**MeIm** = 1:3 experiments, 62.0 mg of basic zinc carbonate (0.113 mmol, 0.57 mmol of Zn) and 140.4 mg of 2-methylimidazole (1.71 mmol) were used.

To study the effect of different added liquids on the rate of carbonate formation, liquid assisted grinding (LAG) experiments were conducted where 75 μL of a given liquid (methanol, ethanol, isopropanol, or water) were added to the reaction mixture. Additionally, ion-assisted neat grinding (ING) and ion- and liquid-assisted grinding (ILAG) experiments were conducted where 10.5 mg (0.13 mmol, 22.5 mol% compared to Zn) of ammonium nitrate was added in the reaction mixture. In the ILAG experiment, an additional 75 μL of water was also added.

In all cases, FT-IR and PXRD measurements were performed without washing the samples.

As in large-scale milling reactions, the small-scale LAG reactions in a Zn:H**MeIm** ratio of 1:2 using methanol or ethanol initially show only the formation of the desired ZIF8 product (5, and 10 min millings, Figure S21). However, after 30 min milling, both sets of reactions show the formation of the zinc carbonate methylimidazolate byproduct, with the ethanol-assisted reaction seemingly resulting in the formation of a smaller amount of byproduct. This is in line with the hypothesis that blocking the pores of the newly formed ZIF8 would also block the byproduct formation; ethanol molecules have a larger volume and diameter, so they could block the pores more effectively. The IR experiments conducted on experiments with added ethanol show that it is indeed present in the reaction mixture (Figure S24), though it is impossible to say if it is adsorbed on the surface, or absorbed inside the pores. Furthermore, as the ZIF8 framework is hydrophobic, it is likely that larger, less polar liquid molecules are better guests for the framework, and hence more efficient in blocking the framework pores.

To further test the limits of the hypothesis, experiments with water, a very small and very polar potential guest, and isopropanol, a much larger (and much less polar) potential pore-blocker, were conducted (Figure S22). Based on the PXRD results, isopropanol indeed prevented the byproduct formation much better than water (only a very small amount of **1** was formed, even after 30 min milling), further reinforcing the hypothesis.

Another important factor in byproduct formation is the speed of the reaction. It appears that the byproduct is not formed simultaneously with the desired product, ZIF8. Instead, ZIF8 is formed first, and then transforms to the byproduct over time. It follows that slower reactions will produce less of the byproduct in a giver timeframe, while faster reactions will produce more of it. Indeed, the small-scale NG reaction doesn't seem to produce any **1** after 30 min, LAG reactions produce mostly ZIF8, with some amount of **1** dependent on the milling liquid, while the fast ING and ILAG reactions provide full conversion into the byproduct **1** with no signs of ZIF8 in only 30 min (Figure S22).

Finally, performing the LAG synthesis with basic zinc carbonate and 2-methylimidazole in a 1:3 ratio provided only ZIF8 after 30 min milling, with no carbonate byproduct formation, no matter if methanol, ethanol, or isopropanol were used as LAG liquids (Figure S23).

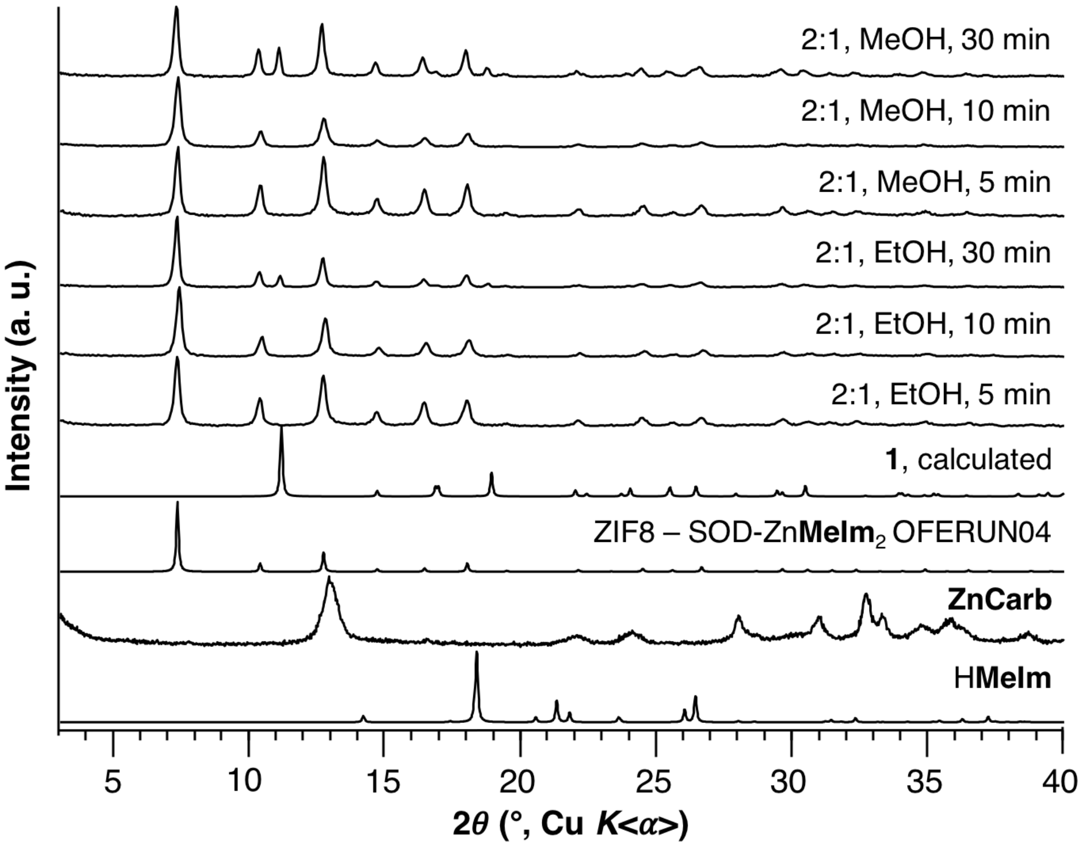

Figure S21. Comparison of PXRD patterns of reagents and products of small scale LAG reactions of basic zinc carbonate and 2-methylimidazole (Zn:H**MeIm** stoichiometric ratio of 1:2) in the presence of ethanol or methanol, after 5, 10, or 30 minutes of milling. Simulated PXRD patterns are shown for of SOD-Zn**MeIm**$_2$ and the complex zinc carbonate methylimidazolate **1**.

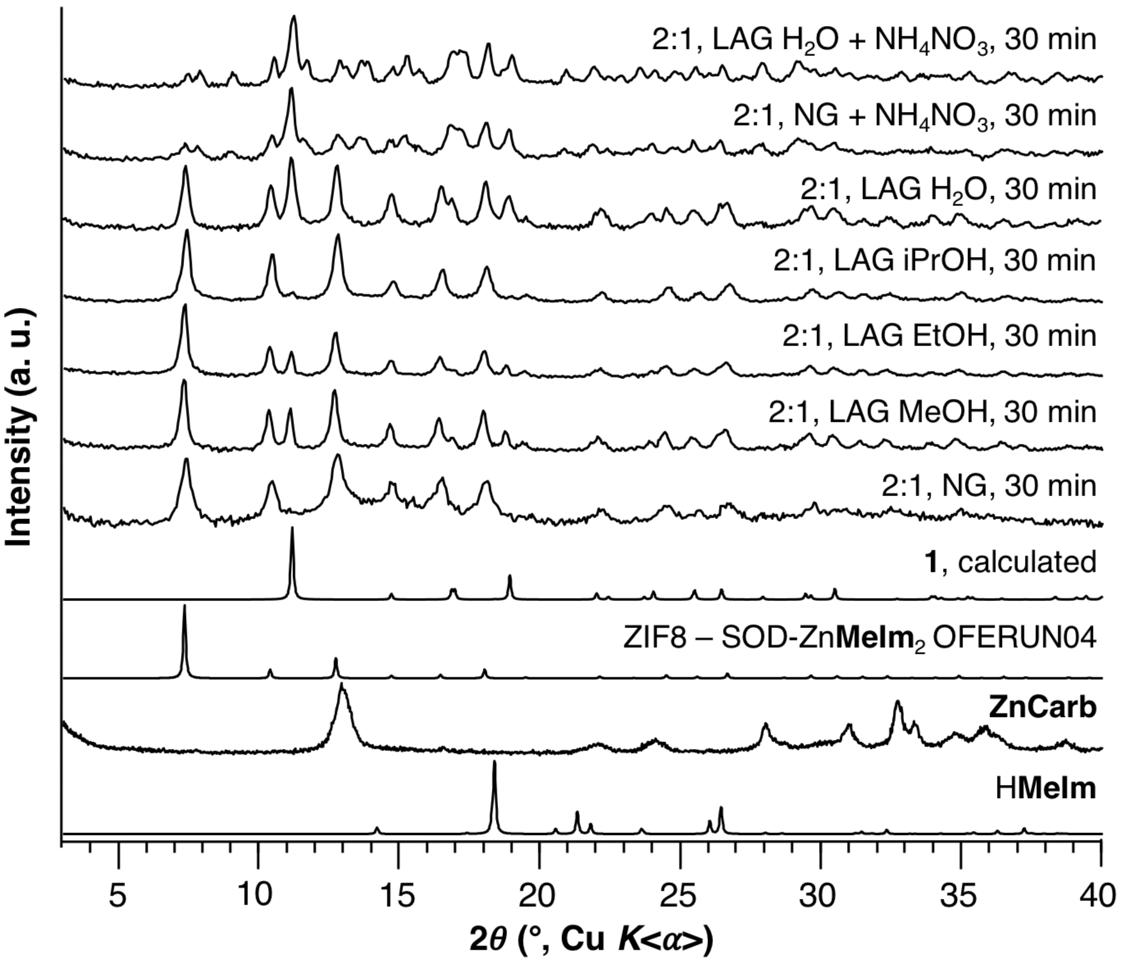

Figure S22. Comparison of PXRD patterns of reagents and products for small scale LAG reactions of basic zinc carbonate and H**MeIm** in the respective stoichiometric ratio of Zn to H**MeIm** of 1:2, using methanol, ethanol, isopropanol or water as milling liquids after 30 minutes of milling. PXRD patterns of the products of ion-assisted neat grinding (ING, NH_4NO_3 additive) and ion- and liquid-assisted grinding (ILAG, NH_4NO_3 and H_2O additives) reactions of basic zinc carbonate and 2-methylimidazole (Zn:H**MeIm** = 1:2) after 30 min of milling. Simulated PXRD patterns of SOD-Zn**MeIm**$_2$ and complex zinc carbonate methylimidazolate **1**.

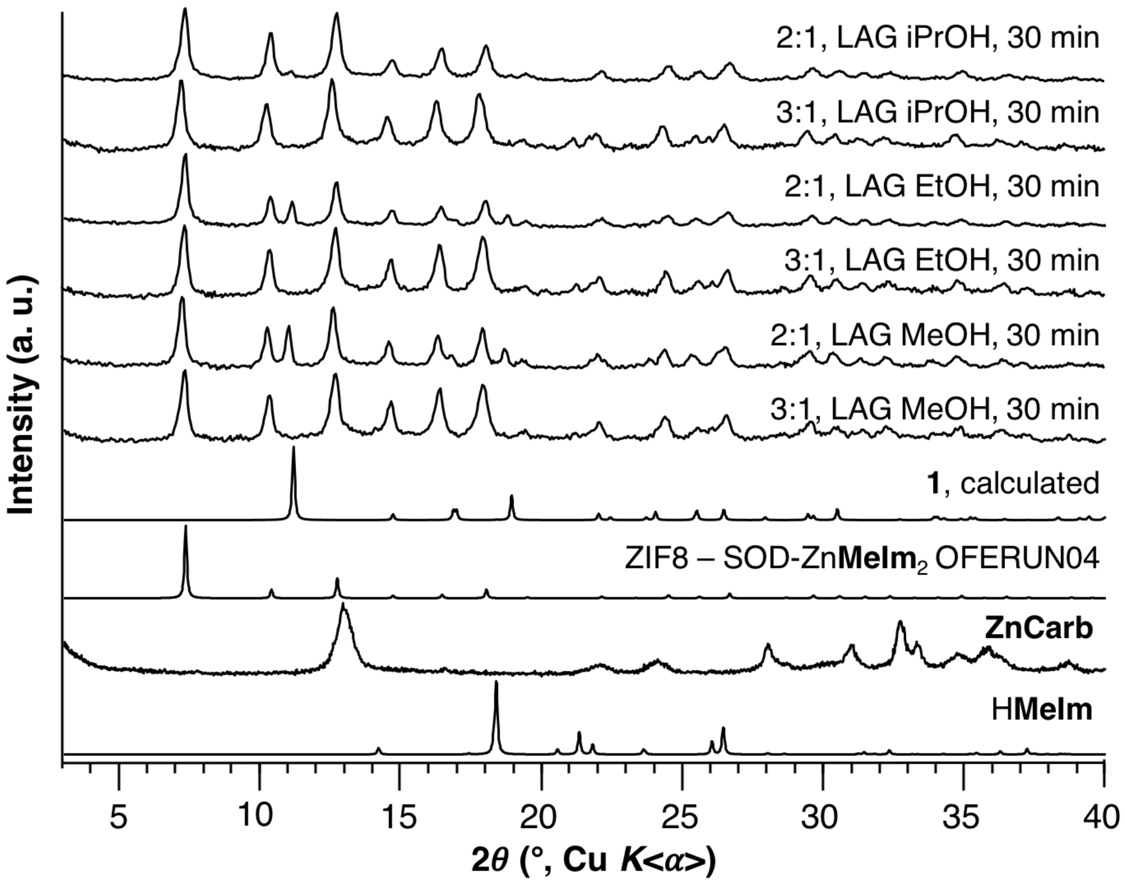

Figure S23. Comparison of PXRD patterns of reagents and products of small scale LAG reactions of basic zinc carbonate and 2-methylimidazole (stoichiometric ratio of zinc to H**MeIm** is 1:2 or 1:3) in the presence of methanol, ethanol, and isopropanol, after 30 min of milling. Simulated PXRD patterns of SOD-Zn**MeIm**$_2$ and complex zinc carbonate methylimidazolate **1**.

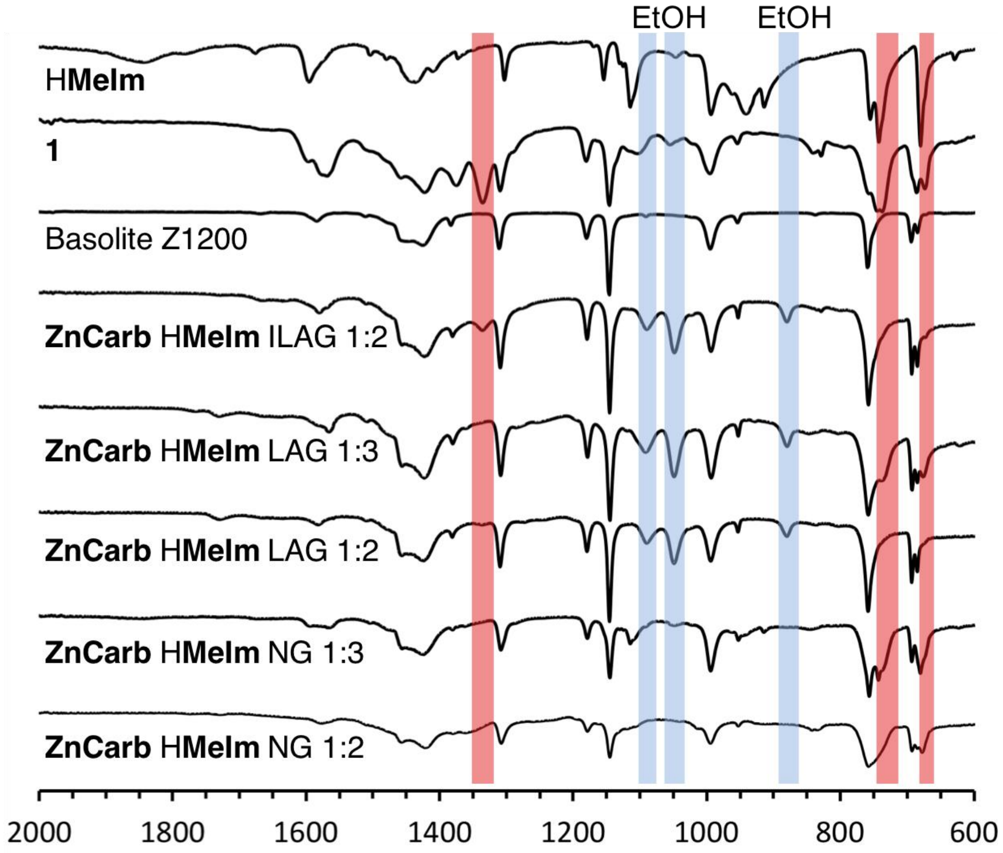

Figure S24. Comparison of FT-IR spectra of small-scale SOD-Zn**MeIm**$_2$ milling syntheses from zinc carbonate and 2-methylimidazole in a stoichiometric ratio of zinc to H**MeIm** of 1:2 (NG, LAG, and ILAG) and 1:3 (NG and LAG) after 30 min of milling. Blue highlights represent the peak positions of ethanol, while red highlights represent the peak positions of **1**.

2.5. Large scale solvent-free synthesis and purification of SOD-Zn(MeIm)$_2$

A 90 g scale solventless synthesis of ZIF-8 was performed using the Zn:H**MeIm** ratio of 1:3. Basic zinc carbonate (43.8 g, 0.08 mol = 0.40 mol Zn) and 2-methylimidazole (98.4 g, 1.20 mol) were milled in a 250 mL steel jar with 2 large stainless steel balls, using a PM 100 planetary mill, for a total of 165 minutes (4 x 30 min + 45 min). 5 g of the resulting ZIF-8 were placed in a 25 mL round bottom flask. The flask was heated to 200 °C under vacuum for 5 hours using Schlenk line techniques.
The purified sample was characterised by PXRD (Figure S25) and TGA (Figure S26), and confirmed to be ZIF-8.

PXRD analysis confirmed the formation of ZIF-8 by NG and the conservation of crystallinity upon sublimation of the included 2-methylimidazole. The TGA curves shown in Figure S26 highlighted the presence of included H**MeIm** in the material before sublimation; corresponding to the weight loss observed between 145 and 245 °C. Additionally, the TGA curve for the ZIF-8 material after sublimation corroborated the successful purification of ZIF-8, as no significant weight loss attributed to unreacted H**MeIm** was observed.

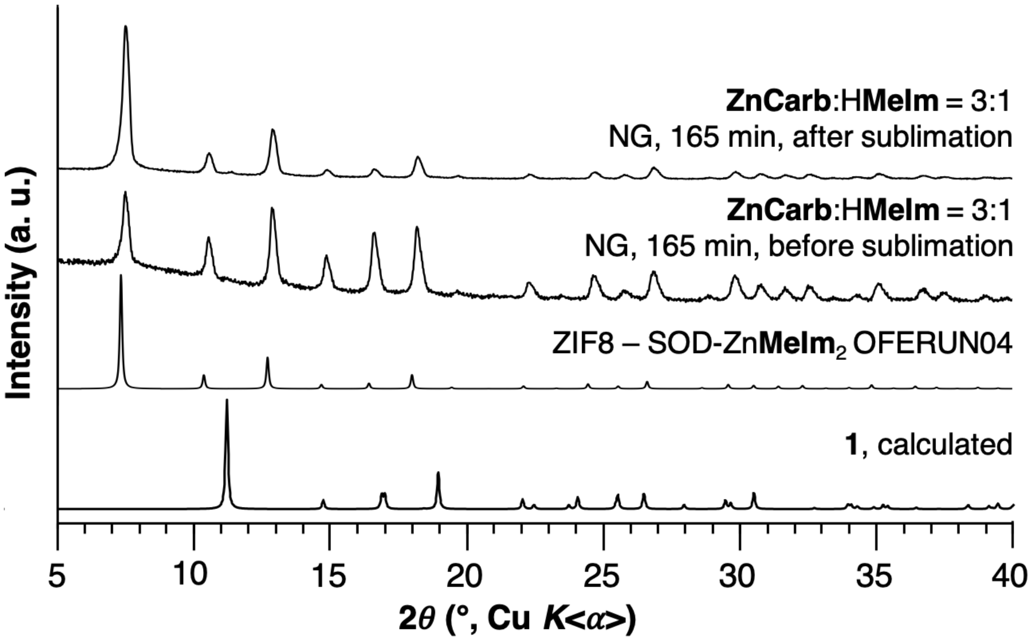

Figure S25. PXRD patterns of the product obtained by large scale NG milling of zinc carbonate and 2-methylimidazole (stoichiometric ratio Zn:H**MeIm** = 1:3), before and after sublimation under vacuum at 200 °C. Simulated PXRD patterns of SOD-Zn(**MeIm**)$_2$ and complex zinc carbonate methylimidazolate **1**.

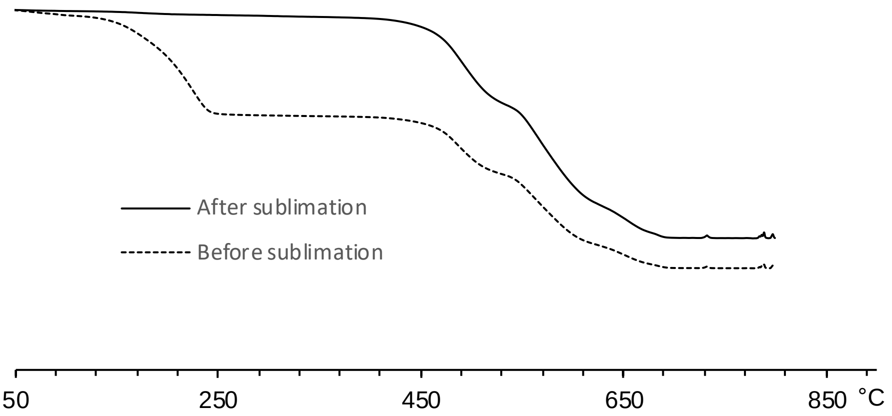

Figure S26. Comparison of TGA curves (measured in a dynamic air atmosphere) of the product from large-scale neat milling of basic zinc carbonate and H**MeIm** for 165 minutes (1:3 stoichiometric ratio of Zn to H**MeIm**), before and after sublimation under vacuum at 200°C.

Finally, low temperature N_2 adsorption isotherms (Figure S27) were used to elucidate the porosity of the ZIF-8 material prepared and purified without using any solvent. A BET surface area of 1785 m^2/g was obtained for the material after sublimation; comparable to the 1758 m^2/g surface area recorded for the commercial Porolite Z8 kindly supplied by MOF Technologies Ltd.

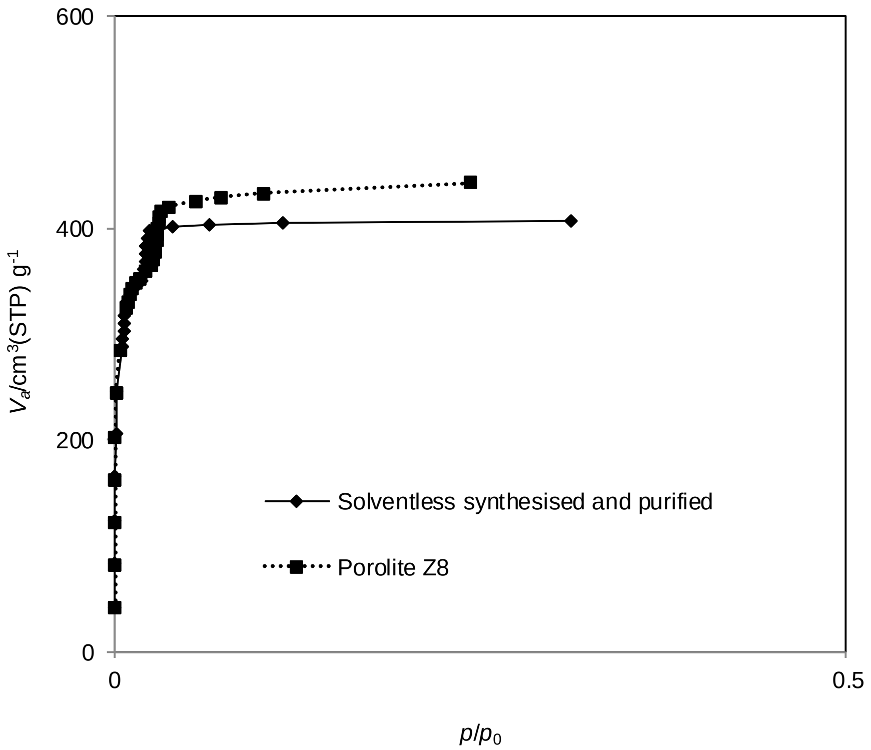

Figure S27. Comparison of N_2 isotherms (measured at 77K) of ZIF-8 (synthesised and purified without solvent) and Porolite Z8 becnhmark material supplied by MOF Technologies.

2.6. Structure determination of compound 1, Zn_2(MeIm)$_2CO_3$, directly from PXRD data

A sample of **1**, Zn_2(**MeIm**)$_2CO_3$), was prepared according to a literature procedure[4], and a high-quality laboratory PXRD pattern suitable for structure determination was recorded as described in Section 1.2. In addition to the major contribution due to **1**, the PXRD data also contained low-intensity peaks due to an impurity amount of ZIF-8.

The peaks due to **1** in the PXRD data were indexed using the ITO code in the program CRYSFIRE, giving the following unit cell with orthorhombic metric symmetry: a = 10.54 Å, b = 12.06 Å, c = 4.70 Å (V = 596.5 Å^3). The space group was assigned as *Pba*2. From consideration of density and the volume of the unit cell, the crystal structure has two formula units (Z = 2) of Zn_2(**MeIm**)$_2CO_3$ in the unit cell. Profile fitting using the Le Bail method in the GSAS program (with the ZIF-8 impurity included as a second phase in the profile fitting calculation) gave a good quality of fit (R_{wp} = 3.99%, R_p = 2.81%; Figure S28). The refined unit cell and profile parameters for **1** were used in the subsequent structure solution calculation.

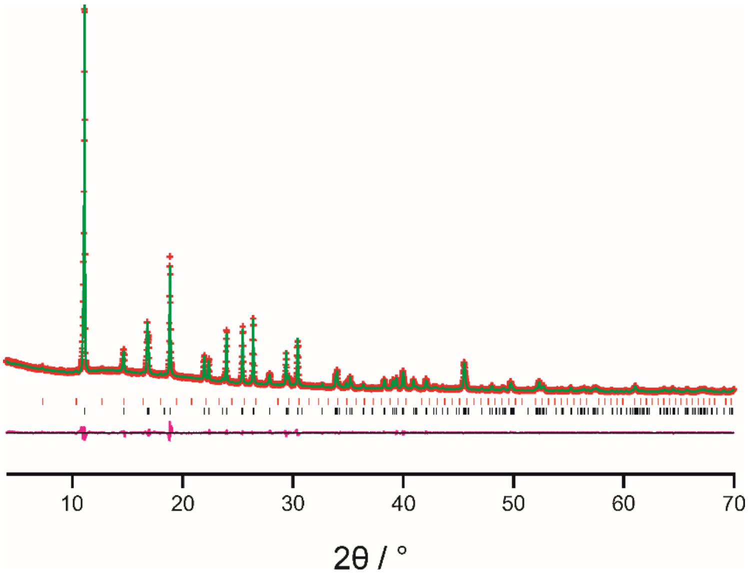

Figure S28. Le Bail profile fit of the PXRD data for **1**, Zn_2(**MeIm**)$_2CO_3$ [red "+" signs, experimental data; green line, calculated data; magenta line (at bottom), difference plot; black tick marks, predicted peak positions for Zn_2(**MeIm**)$_2CO_3$; red tick marks, predicted peak positions for ZIF-8].

Structure solution was carried out using the direct-space genetic algorithm (GA) technique in the program EAGER. As there are two formula units of Zn_2(**MeIm**)$_2CO_3$ in the unit cell, the composition of the asymmetric unit is Zn(**MeIm**)$(CO_3)_{0.5}$. In the GA structure solution calculation, the asymmetric unit was defined by three structural fragments: (a) a fragment representing the zinc cation, defined by three positional variables; (b) a fragment representing the methylimidazolate anion, defined by three positional variables and three orientational variables; and (c) a fragment representing one half carbonate anion, comprising one carbon atom with half occupancy, one oxygen atom with half occupancy and one oxygen atom with full occupancy – one C–O bond of this fragment (involving the

carbon and oxygen atoms with half occupancy) was located on a 2-fold rotation axis parallel to the *c*-axis, and the fragment was allowed to translate along this axis and to rotate around this axis.

In total, 16 independent GA structure solution calculations were carried out. Each calculation involved the evolution of 100 generations for a population of 100 structures, with 10 mating operations and 50 mutation operations per generation. The same structure solution of highest quality (corresponding to the lowest value of Rwp) was obtained in all 16 cases.

The structure solution was then used as the starting model for Rietveld refinement, carried out using the GSAS program, with the ZIF-8 impurity included as a second phase in the refinement. In the Rietveld refinement, standard restraints were applied to bond lengths and bond angles, and planar restraints were applied to the methylimidazolate group. The final Rietveld refinement gave a good quality of fit to the PXRD data (R_{wp} = 4.97%, R_p = 3.37%; Figure S28), with the following refined parameters: a = 10.52947(26) Å, b = 12.0539(4) Å, c = 4.69283(11) Å, V = 595.62(4) Å^3 (2θ range, 4 – 70°; 3866 profile points; 61 refined variables).

2.7. Cobalt(II) carbonate and imidazole experiments

In a typical large-scale reaction, 7.0 g of cobalt(II) carbonate (0.059 mol) and 8.0 g of imidazole (0.118 mol) were milled in a 250 mL steel jar with 7 large balls, using a PM 400 planetary mill at a frequency of 300 rpm for 90 min. For liquid assisted grinding (LAG) experiments 2 mL of methanol were added to the reaction mixture. The pressure and temperature in the jars were measured during milling (Fig S28). The samples were analyzed *via* PXRD (Fig S29)

Both the NG and LAG syntheses appeared to be much slower than the analogous reactions using basic zinc carbonate, and were not equilibrated even after 90 min (Fig S29). The NG reaction showed a maximum in pressure around 85 min, followed by a pressure decrease, similar to the reactions involving basic zinc carbonate and 2-methylimidazole. The LAG reaction showed a continuous increase in pressure and achieved a much higher final pressure than the NG reaction

The NG synthesis resulted in the formation of a predominantly amorphous product (Figure S30) with a pressure yield of 42.4%. The LAG synthesis resulted in the formation of a mixture of products including **cag-**Co**Im**$_2$ (most likely as a methanol solvate), **zni-**Co**Im**$_2$, and a yet not identified phase characterized by a signal at 2θ of ca. 23.5° (Figure S29), with a pressure yield of 91.5%.

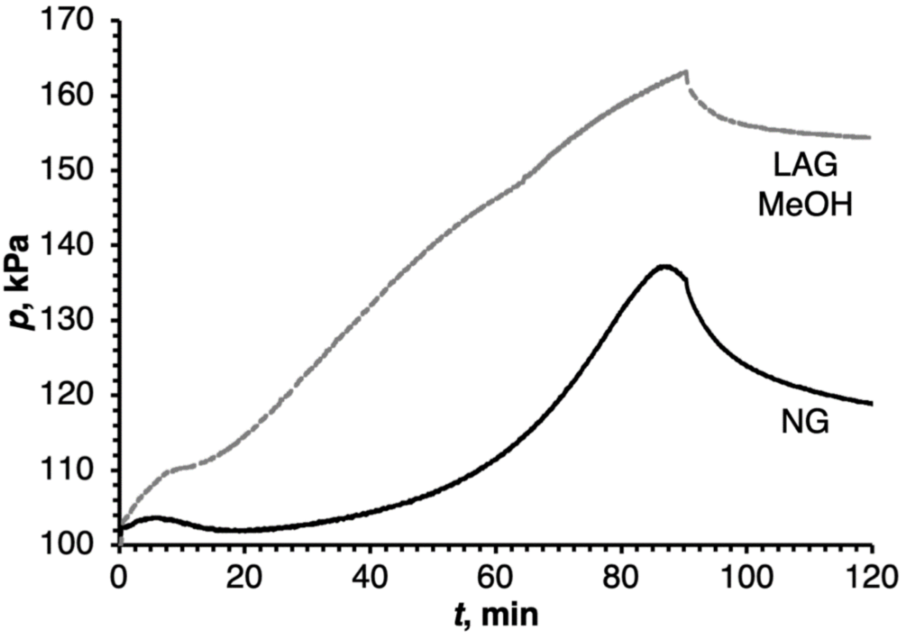

Figure S29. Gas pressure measurements for the neat grinding (NG, black, solid), and liquid-assisted grinding (LAG, grey, dashed) reactions of cobalt(II) carbonate and imidazole. The milling liquid for LAG experiment was methanol, and the reaction time was 90 min.

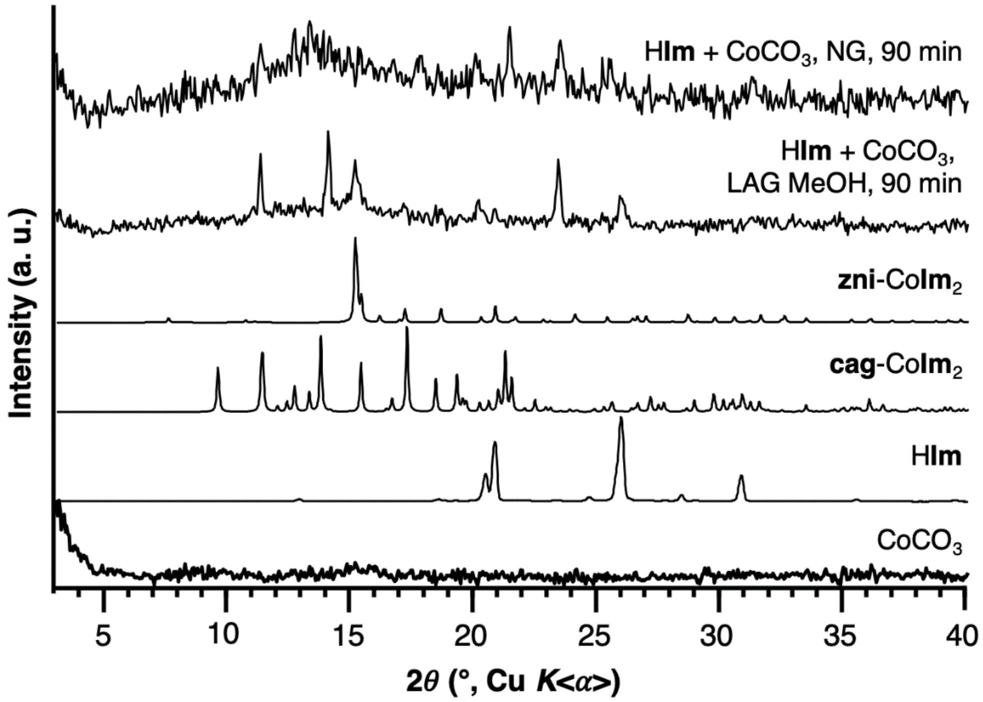

Figure S30. Comparison of the PXRD patterns for the products of the large-scale milling reactions (reaction time of 90 min) of cobalt(II) carbonate and imidazole using NG and LAG, as well as the PXRD patterns of the starting reagents. The analysis of the product mixtures is made difficult by X-ray fluorescence of cobalt-based samples when using CuK_α radiation.

3. References

[1] O.C. Bridgeman and E.W. Aldrich, *J. Heat Transfer*, 1964, **86**, 279-286.

[2] D. Ambrose, C.H.S. Sprake and R. Townsend, *J. Chem. Thermodyn.*, 1975, **7**, 185-190.

[3] X. Gui, Z. Tang and W. Fei, *J. Chem. Eng. Data*, 2011, **56**, 2420–2429.

[4] C. Mottillo and T. Friscic, *Angew. Chem., Int. Ed.*, 2014, **53**, 7471.

4. Author Contributions

IB performed all PXRD, SSNMR, TGA, IR, and milling experiments, as well as data analysis, figure and Supplementary Information preparation, under the supervision of TF and KTH. WY, YL and CM performed preliminary mechanochemical experiments involving basic zinc carbonate and imidazoles. CM and YL prepared **1** for PXRD structure solution. CM assisted IB in SSNMR data collection. YZ and JC performed large scale solventless milling experiments and purification under the supervision of SLJ. FD, PAW and KDMH performed the crystal structure determination of **1** from PXRD data. IB and TF wrote the manuscript, with input from KDMH, SLJ, and KTH. Project was originally conceived, and funding secured by TF and SLJ.
